# The aspartic proteinase family of three *Phytophthora *species

**DOI:** 10.1186/1471-2164-12-254

**Published:** 2011-05-20

**Authors:** John Kay, Harold JG Meijer, Arjen ten Have, Jan AL van Kan

**Affiliations:** 1 School of Biosciences, Cardiff University, Cardiff CF10 3AX, UK; 2 Laboratory of Phytopathology, Wageningen University, P.O. Box 8025, 6700EE Wageningen, The Netherlands; 3 Instituto de Investigaciones Biologicas - CONICET, Facultad de Ciencias Exactas y Naturales, Universidad Nacional de Mar del Plata, CC1245, 7600 Mar del Plata, Argentina

## Abstract

**Background:**

*Phytophthora *species are oomycete plant pathogens with such major social and economic impact that genome sequences have been determined for *Phytophthora infestans*, *P. sojae *and *P. ramorum*. Pepsin-like aspartic proteinases (APs) are produced in a wide variety of species (from bacteria to humans) and contain conserved motifs and landmark residues. APs fulfil critical roles in infectious organisms and their host cells. Annotation of *Phytophthora *APs would provide invaluable information for studies into their roles in the physiology of *Phytophthora *species and interactions with their hosts.

**Results:**

Genomes of *Phytophthora infestans*, *P. sojae *and *P. ramorum *contain 11-12 genes encoding APs. Nine of the original gene models in the *P. infestans *database and several in *P. sojae *and *P. ramorum *(three and four, respectively) were erroneous. Gene models were corrected on the basis of EST data, consistent positioning of introns between orthologues and conservation of hallmark motifs. Phylogenetic analysis resolved the *Phytophthora *APs into 5 clades. Of the 12 sub-families, several contained an unconventional architecture, as they either lacked a signal peptide or a propart region. Remarkably, almost all APs are predicted to be membrane-bound.

**Conclusions:**

One of the twelve *Phytophthora *APs is an unprecedented fusion protein with a putative G-protein coupled receptor as the C-terminal partner. The others appear to be related to well-documented enzymes from other species, including a vacuolar enzyme that is encoded in every fungal genome sequenced to date. Unexpectedly, however, the oomycetes were found to have both active and probably-inactive forms of an AP similar to vertebrate BACE, the enzyme responsible for initiating the processing cascade that generates the Aβ peptide central to Alzheimer's Disease. The oomycetes also encode enzymes similar to plasmepsin V, a membrane-bound AP that cleaves effector proteins of the malaria parasite *Plasmodium falciparum *during their translocation into the host red blood cell. Since the translocation of *Phytophthora *effector proteins is currently a topic of intense research activity, the identification in *Phytophthora *of potential functional homologues of plasmepsin V would appear worthy of investigation. Indeed, elucidation of the physiological roles of the APs identified here offers areas for future study. The significant revision of gene models and detailed annotation presented here should significantly facilitate experimental design.

## Background

The oomycete genus *Phytophthora *is comprised of over 100 species, most of which are plant pathogens [1]. The most renowned and devastating species is *P. infestans*, which contributed to the potato famine in Ireland, resulting in a million deaths through starvation, and the mass exodus of many more to other locations, principally in North America [2]. The pathogen continues to blight modern agriculture and causes an annual loss in worldwide potato crops approaching $7 billion [3]. The economic damage has intensified research to increase our knowledge of this organism that has now been under investigation for more than 150 years. The genome sequences of *P. infestans*, and two related species, *P. sojae*, causing soybean root and stem rot and *P. ramorum*, the sudden oak death pathogen, were recently determined [4-6]. At 240, 95 and 65 megabases respectively, there is a considerable difference in the genome sizes of *P. infestans, P. sojae *and *P. ramorum*. Gene numbers among the three genomes are not drastically different and there is considerable co-linearity between them. Genome size expansion in *P. infestans *has mainly resulted from escalation in the number of repeat regions and transposons [4]. Analyses of *Phytophthora *genomes have identified genes encoding proteins containing novel combinations of previously identified domains [4,6-9].

In common with other organisms for which genome sequences have been elucidated, one protein category which is highly represented in the three *Phytophthora *genomes is that of proteolytic enzymes [4]. These protein hydrolases have a wide variety of roles in all organisms including nutrient provision, stress responses and cell death processes. Proteinases are categorised into a number of catalytic types, dependent on the nature of the nucleophile that participates in the catalytic reaction. The MEROPS database (http://merops.sanger.ac.uk) classifies proteinases into clans which contain all the proteins that have arisen from a single evolutionary origin [10]. A clan represents one or more families of proteinases that reveal their evolutionary relationship through similarities at the primary and 3D-structural levels. Our interests over many years have focussed on aspartic proteinases, which belong to the AA clan. Within this clan, most members reside in the A01 family and are related to the archetypal patriarch, pepsin, or in family A02 which encompasses proteinases from retroviruses including HIV [11]. APs participate in a variety of physiological and pathological processes [11] in vertebrates (e.g. renin in hypertension and the β-secretase or BACE in Alzheimer's Disease [12]) while in plants APs have roles in senescence, stress responses and fertilization [13] as well as in defence against pathogens [14]. Reciprocally, APs are deployed by pathogens to facilitate infection e.g. HIV/AIDS [15], thrush (candidiasis) and malaria [16]. Inhibitors targeted against HIV-proteinase are now in widespread use in therapy for treatment of HIV-infected individuals [17]. Recently it has been shown that one AP of *Plasmodium falciparum *is an ideal target for development of therapeutic inhibitors to treat malaria [18,19]. This AP is responsible for processing *Plasmodium *effector proteins in a step that is crucial for their export into the host red blood cell, thereby reprogramming the host cell to meet the demands of the parasite.

With these examples serving to illustrate the reliance of both host and pathogens on AP activities, it was of considerable interest to establish and annotate the complement of APs encoded within the three *Phytophthora *genomes, particularly since one of the major current subjects of oomycete research is on effectors that modulate host cell physiology and immune responses. *Phytophthora *effectors are small, secreted proteins that undergo translocation into host cells [20] in a manner analogous to that for the *Plasmodium *effectors [18,19]. However, the components of the *Phytophthora *translocation machinery remain to be identified. Here we describe the complete AP family of three *Phytophthora *species and identify some of its members which may participate in effector translocation.

## Results and Discussion

Genome sequences of *P. infestans *(available through the Broad Institute website http://www.broadinstitute.org/annotation/genome/phytophthora_infestans/MultiHome.html), *P. sojae *and *P. ramorum *(from JGI at http://genome.jgi-psf.org/) were analysed for the presence of genes encoding putative pepsin-like aspartic proteinases as described in the Methods. Pepsin-like APs from family A01 are typically produced in the form of a precursor, consisting of a signal peptide followed by a propart region that has to be excised from the zymogen polypeptide chain in order to generate the mature enzyme. The mature enzyme region consists of two homologous catalytic domains, each of which provides a catalytic Asp residue to the active site [11,21]. Each Asp residue is located within the hallmark motif Asp-Thr/Ser-Gly, which is followed by a hydrophobic-hydrophobic-Gly sequence, as exemplified by the family patriarch, pig pepsin (Additional File 1). Together, these motifs form a structural feature known as a psi loop [22]. A Tyr residue is located at position 75 (pepsin numbering) in a β-hairpin loop which overlies the active site. This residue is strictly conserved in all active pepsin-like APs and serves as a landmark residue that can be anticipated to be present in newly-identified AP sequences. A number of other motifs and landmark residues (including Trp 39 and several intramolecular disulphide bonds - Additional File 1) are present at characteristic locations along A01 family member polypeptides.

A total of 11 predicted protein sequences exhibiting some or all of these hallmark features and landmark residues were identified in *P. infestans*, with 12 and 14 entries detected in *P. sojae *and *P. ramorum*, respectively. Inspection of the sequences revealed that, in *P. ramorum*, gene model Pr_73565 was erroneous due to incorrect assembly of the DNA sequence in this region and gene model Pr_84645, present in a correctly assembled sequence, was a pseudogene lacking at least 250 residues of the N-terminus of a functional AP. In *P. sojae*, the sequence encoded by gene Ps_129567 was highly similar to APs but the two hallmark Asp-Thr/Ser-Gly motifs were replaced by Asp-Val-Met and Phe-Thr-Met respectively, such that the encoded protein could not possibly be active. This entry, which lacked an orthologue in both *P. infestans *and *P. ramorum*, was thus considered also to be a pseudogene. The above three gene models were thus excluded from further analyses.

The remaining 34 genes were the only ones consistently identified by repeated search using PSI-Blast [23] or HMMer [24], so that it would appear no further AP homologues are present within these three genomes. These 34 gene models were analysed (Table 1) and are further discussed in groups on the basis of orthology. Comparison of the orthologues from the three species revealed in a number of cases that the gene models, as originally annotated (Table 1), were likely to be incorrect. The wealth of information available on pepsin-like AP sequences (http://merops.sanger.ac.uk) including the hallmark motifs/landmark residues described above, was applied together with available EST and trace file data, to guide the final gene predictions for putative APs encoded in the three *Phytophthora *genomes. Additional File 2 lists the gene models that required correction. More than half of the original gene models annotated in the *P. sojae *and *P. ramorum *databases were correct, whilst almost all of the original entries for *P. infestans *were erroneous. The annotation errors that were identified (Additional File 2) included mis-identification (i) of the initiator Met residue, (ii) of intron presence/absence, (iii) of the 5' and/or 3' splice junctions of introns as well as (iv) sequence trace mis-reads or (v) trace mis-usage in assembly of the *P. infestans *sequence due to heterozygosity. Annotation errors in two *P. infestans *genes due to sequencing related issues were corrected by re-examination of original sequence trace files at NCBI and by re-sequencing relevant regions. The corrected nucleotide sequences of these genes (PITG_08190 and PITG_09387) are deposited in NCBI under accession numbers HM588685 and HM588686, respectively. For all gene models, the intron positions were verified by inspection of EST information, if available, and by comparison with DNA sequences of the orthologous gene in the other two species for the presence or absence of possible splice junctions at the equivalent nucleotide position(s). The corrected gene models and their encoded polypeptides are summarised in Table 2. All corrections proposed for the *P. infestans *gene models were confirmed by available EST data or by cloning and sequencing the cDNA. Two of the three proposed corrected gene models for *P. sojae *(Ps_157552, Ps_158877) were confirmed by cloning and sequencing the cDNA, while for the third (Ps_135764), correction could not be confirmed as cDNA amplification was unsuccessful. Corrections in *P. ramorum *gene models were not experimentally validated as this species has a quarantine status. An initial insight into the expression patterns of these APs was obtained from microarray analysis of *P. infestans *genes using the genome-wide NimbleGen array [4]. Consistent levels of expression were observed for each of the eleven PiAP genes during culture on different agar media and during infection on potato (Additional File 3).

**Table 1:** Aspartic Proteinase Genes in *Phytophthora *spp.

*Phytophthora infestans*			*Phytophthora sojae*			*Phytophthora ramorum*		
**PITG accession number**	**Predicted intron number**	**Predicted length (residues)**	**Ps accession number**	**Predicted intron number**	**Predicted length (residues)**	**Pr accession number**	**Predicted intron number**	**Predicted length (residues)**

09387	2	815	109366	0	393	72239	0	389
12357	0	469	144332	0	472	84847	0	469
-	-	-	157552	5	379	77872	8	698
05114	2	545	132274	1	573	82845	1	548
10524	0	737	138576	0	751	84399	0	753
04975	1	512	136075	0	587	73566	0	582
05004	3	307	-	-	-	73546	0	432
08190	1	556	135399	0	585	78678	0	611
06900	5	479	135764	3	627	79884	4	644
02623	2	681	144093	2	719	77495	2	636
02624	2	350	144091	11	806	77493	3	360
11522	1	446	141367	0	531	74839	0	525

The number of introns and length of each polypeptide predicted by the database-annotated gene models (January 2010) are depicted for the orthologues from *P. infestans*, *P. sojae *and *P. ramorum *set out in each row.

**Table 2:** Corrected Aspartic Proteinase gene models in *Phytophthora *spp.

	*Phytophthora infestans*			*Phytophthora sojae*			*Phytophthora ramorum*		
	**Gene model**	**#introns**	**Length**	**Gene model**	**#introns**	**Length**	**Gene model**	**#introns**	**Length**

PxAP1	PITG_09387	0	390	Ps_109366	0	393	Pr_72239	0	389
PxAP2	PITG_12357	0	472	Ps_144332	0	472	Pr_84847	0	469
PxAP3	none			Ps_157552	4	425	Pr_77872	4	414
PxAP4	PITG_05114	1	559	Ps_132274	1	573	Pr_82845	1	548
PxAP5	PITG_10524	0	737	Ps_138576	0	751	Pr_84399	0	753
PxAP6	PITG_04975	0	566	Ps_136075	0	587	Pr_73566	0	582
PxAP7	PITG_05004	0	444	none			Pr_73546	0	432
PxAP8	PITG_08190	0	574	Ps_135399	0	585	Pr_78678	0	586
PxAP9	PITG_06900	4	647	Ps_135764	4	654	Pr_79884	4	644
PxAP10	PITG_02623	2	681	Ps_144093	2	719	Pr_77495	2	650
PxAP11	PITG_02624	4	638	Ps_144091	4	642	Pr_77493	4	640
PxAP12	PITG_11522	0	522	Ps_141367	0	531	Pr_74839	0	525

Genes are presented as groups of orthologues. The generic descriptors PxAP1-12 are used to denote the respective encoded polypeptides. The number of residues in each polypeptide is indicated.

The final protein sequences predicted for each of the 34 APs from the three *Phytophthora *species are listed individually in Additional File 4. Each of these sets of orthologous polypeptides was examined for the presence/absence of a signal peptide and a propart segment preceding the mature enzyme region. These features are summarised in Table 3. The individual signal peptide/propart segment sequences for each of the 12 sets of orthologues are aligned in Additional File 5. In order to simplify the descriptions that follow, the identifiers PxAP1-12 were assigned to each of these sets of orthologues.

**Table 3:** Component segments flanking the N and C-terminal ends of the mature enzyme regions of the *Phytophthora *Aspartic Proteinases.

	Signal Peptide	Pro-Peptide	C-terminal extension	Number of Trans-membrane domains
PxAP1	**+**	**+**	-	0
PxAP2	-	**+**	**+**	1
PxAP3	**+**	**+**	**+**	0
PxAP4	**+**	**+**	**+**	1
PxAP5	**+**	**+**	**+**	7
PxAP6	**+**	-	**+**	1
PxAP7	**+**	-	**+**	1
PxAP8	**+**	-	**+**	1
PxAP9	**+**	-	**+**	1
PxAP10	**+**	**+**	**+**	1
PxAP11	**+**	**+**	**+**	1
PxAP12	**+**	**+**	**+**	1

Except for PxAP1, the polypeptides continued beyond the conventional location of the C-terminus of an archetypal AP, as defined by the reference standard, pig pepsin (Additional File 1). Each of the PxAP2 - PxAP12 polypeptides thus contained a C-terminal extension (Table 3). These extensions differed markedly in length and sequence (Additional File 6) and could only be aligned with the sequences of their orthologue(s) from the other *Phytophthora *species, and not with any other sequence in the set of entries or with any archetypal AP. Only short extensions were present in the PsAP3 and PrAP3 polypeptides (Additional File 6). The C-terminal extensions for PxAP2 and PxAP4 - PxAP12 were considerably longer and all contained at least one stretch of approximately 20 consecutive hydrophobic residues (marked in Additional File 6 by a box) which are predicted to provide a membrane-spanning segment. Thus, almost all the *Phytophthora *APs (PxAP2 and PxAP4 - PxAP12) are predicted to be membrane-bound enzymes. While it is not uncommon for individual APs from other species to be membrane-anchored in such a fashion (e.g. human BACE, plasmepsin V from *Plasmodium falciparum*), it is unprecedented for almost the entire AP complement of an organism to appear to be membrane-bound enzymes. Considerable experimental effort will need to be invested to establish whether all of these predicted polypeptides are indeed membrane-associated but in previous investigations, APs that have been predicted to contain a membrane-associated segment have always been proven by subsequent experimental investigations to be membrane-attached. Further consideration will be given to this aspect for the individual cases described below.

**Figure 1:**
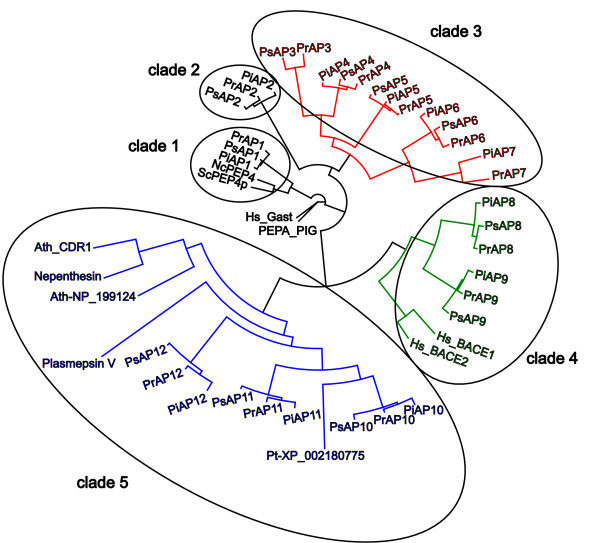
**Phylogenetic tree of aspartic proteinases (AP) from *Phytophthora infestans *(Pi), *Phytophthora sojae *(Ps) and *Phytophthora ramorum *(Pr)**. The tree was generated from sequences of the mature enzyme regions and was rooted at the pig pepsin (PepA_PIG) node. ScPEP4p and NcPEP4 are the respective vacuolar APs from *Saccharomyces cerevisiae *and *Neurospora crassa*. Ath_CDR1 and Ath-NP_199124 are respectively C-terminally unextended and extended APs from *Arabidopsis thaliana*. Pt-XP_002180775 is from *Phaeodactylum tricornutum*. Nepenthesin 1, plasmepsin V and human BACE/BACE2 are as described in the text. Alignment of entries clustering in clades 1 and 2 (in black characters) is shown in Figure 2; alignment of entries clustering in clade 3 (in red characters) is shown in Figure 3; alignment of entries clustering in clade 4 (in green characters) is shown in Figure 4; alignment of entries clustering in clade 5 (in blue characters) is shown in Figure 5.

**Figure 2:**
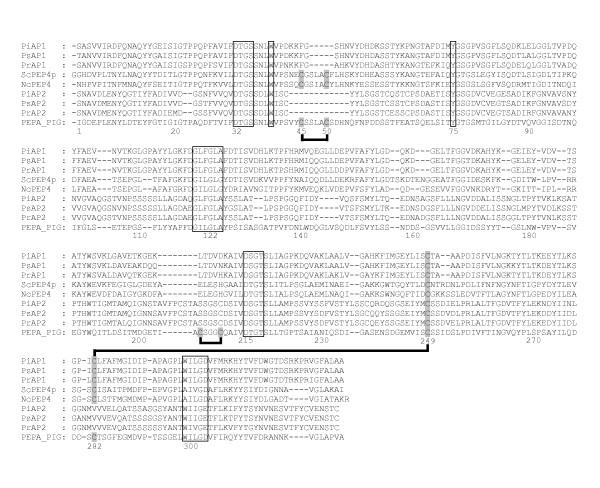
**Alignment of the mature enzyme regions of PxAP1 and PxAP2 predicted polypeptides with pig pepsin**. The numbering system is that of pig pepsin (PepA_PIG) and the vacuolar enzymes from *S. cerevisiae *(ScPEP4p) and *N. crassa *(NcPEP4) are included for comparison. The DT/SG and hydrophobic-hydrophobic-Gly motifs forming the psi loops in each domain are boxed, as are the landmark residues Y75 and W39. The Cys residues contributing the three disulphide bonds common to vertebrate APs such as pig pepsin, are shaded and their pairing is indicated by thick () black lines. Gaps introduced to optimise the alignment are indicated by a hyphen.

**Figure 3:**
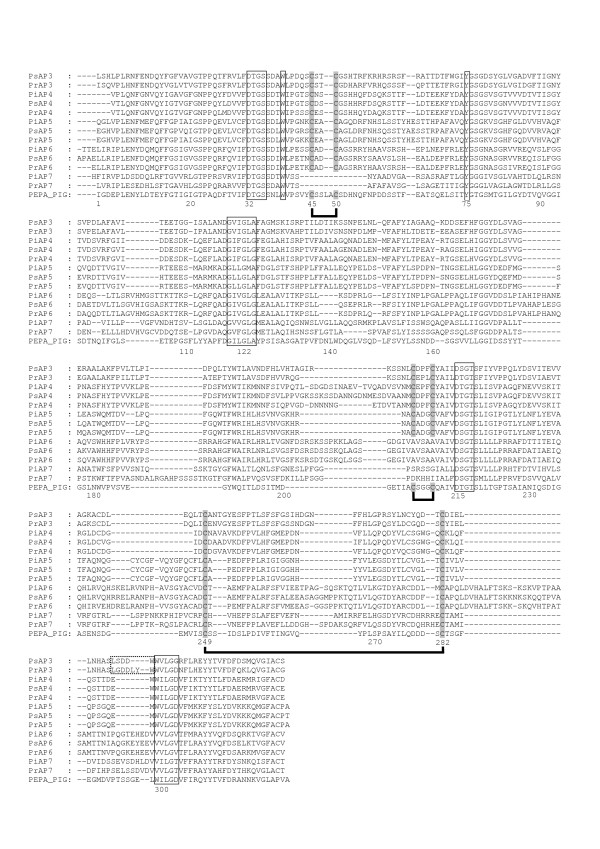
**Alignment of the mature enzyme regions of the PxAP3 - 7 predicted polypeptides with pig pepsin**. The numbering system is that of pig pepsin (PepA_PIG) and other details are as described in the legend to Figure 2. The polyproline loop regions of PsAP3 and PrAP3, discussed in the text, are highlighted by a dotted box ().

**Figure 4:**
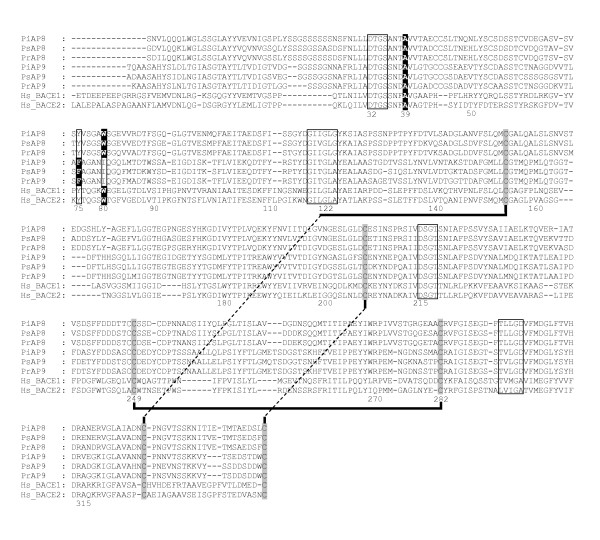
**Alignment of the mature enzyme regions of PxAP8 and PxAP9 predicted polypeptides with human BACE (Hs_BACE1) and BACE2 (Hs_BACE2)**. The numbering system is that of pig pepsin. The DT/SG and hydrophobic-hydrophobic-Gly motifs forming the psi loops in each domain are boxed. Landmark residues 75 and 39 are boxed as in previous figures, but here to emphasise the Y75F and W39A replacements (highlighted black). Residue 80 is also boxed to indicate the presence of a W residue at this location in PxAP8 and BACE/BACE2. The Cys residues contributing the three disulphide bonds in BACE are shaded and their known pairing is indicated by thick () and dotted black lines ().

**Figure 5:**
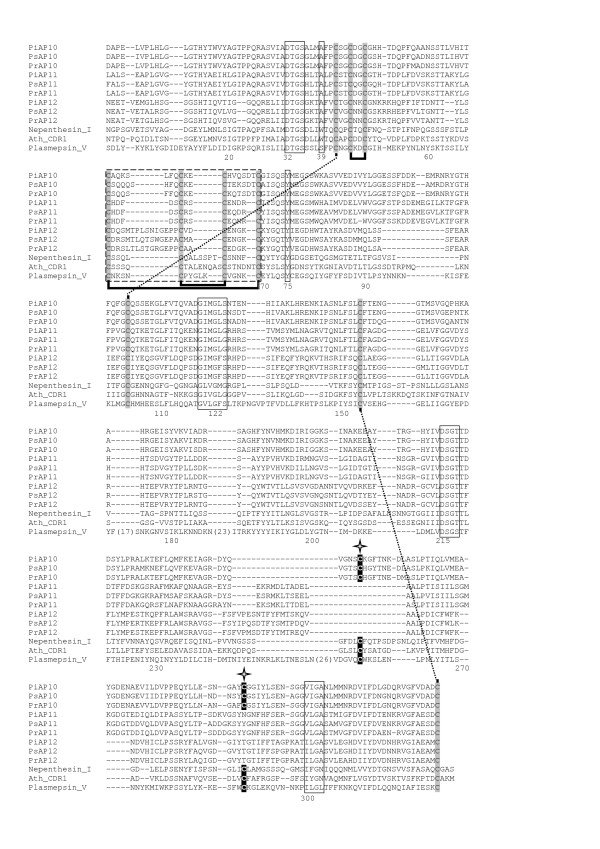
**Alignment of the mature enzyme regions of PxAP10 - 12 predicted polypeptides with representative plant aspartic proteinases and plasmepsin V**. The numbering system is that of pig pepsin. Boxes identifying the hallmark features/landmark residues are as described in the legend to Figure 2. Cys residues and their likely pairing in intramolecular disulphide bonds are indicated by shading and thick black () and dotted () lines, respectively. The two Cys residues which are reported to participate in intermolecular disulphide bonds in Ath_CDR1 are marked with a star (). The region containing the inserted residues of this sub-type of AP is indicated by a dotted box ().

The sequences of the mature enzyme regions of each of the 12 sets of orthologues from the three *Phytophthora *species were aligned and a phylogenetic tree was generated (Figure 1). This resolved the PxAP1-12 sequences into five clades that are related to well-documented APs from other species. Each *Phytophthora *species is thus equipped with an AP gene family, the members of which have distinct gene organisations (Table 3) and encode polypeptides with considerably different sequences (Figures 2, 3, 4, 5) and, potentially, distinct activities. Each of the clades will be considered in turn.

### Archetypal pepsin-like Aspartic Proteinases (MEROPS subfamily A1A)

In clade 1 of the phylogenetic tree (Figure 1), three proteins designated PiAP1, PsAP1 and PrAP1, are encoded by orthologous single exon genes (Table 2). The PxAP1 polypeptides (Table 3) display all of the essential components i.e. signal peptide, propart segment (Additional File 4) and two-domain mature enzyme region (Figure 2), including all the necessary hallmark motifs and residues of archetypal pepsin-like APs described earlier (Additional File 1). These polypeptides are not extended at their C-terminus. The phylogenetic analysis revealed that the three PxAP1 polypeptides cluster closely with fungal APs (Figure 1) that are known to be located in the vacuole of the cell. This subfamily, with saccharopepsin from *S. cerevisiae *as the parent member, is assigned the identifier A01.018 in the MEROPS database. Analyses of all sequenced fungal genomes has revealed that the only AP that is omnipresent is the vacuolar enzyme [25]. Thus, it seems likely that PiAP1, PsAP1 and PrAP1 are the respective vacuolar APs from each *Phytophthora *specie*s*. PiAP1 has been produced in recombinant form and shown to have AP activity on haemoglobin (F. Govers, Wageningen University, personal communication).

The polypeptides of the PxAP2 in clade 2 (Figure 1) are atypical in that they do not contain a signal peptide or a non-classical secretion signal (Table 3 and Additional File 5). This is highly unusual in APs although precedents have been observed in fungi including *Botrytis cinerea *[25] and in bacterial species of marine origin [26]. It is not obvious how these polypeptides are able to become incorporated into a membrane through the (single) hydrophobic segment in their C-terminal extensions (Additional File 6) when there is no signal peptide to mediate transfer into the endoplasmic reticulum. Experiments using fusion constructs with reporter proteins will be necessary to resolve this conundrum.

Within clade 3, five clusters (PxAP3 - PxAP7) were resolved from one another (Figure 1). More profound phylogenetic analysis (not shown) indicates that this clade forms a monophyletic clade with three entries from two filamentous fungi, i.e. the zygomycetes *Rhizopus oryzae *and *Phycomyces blakesleeanus*. PxAP3-encoding genes were only detected in *P. sojae *and *P. ramorum *(gene models Ps_157552 and Pr_77872; Table 1, Table 2). Despite repeated searching, an orthologue of these two in *P. infestans *could not be detected. The PxAP3 genes are located in regions of co-linearity. Scrutiny of the five flanking genes (upstream and downstream) in the *P. sojae *and *P. ramorum *genomes revealed that orthologues are present in similar arrangement in *P. infestans *DNA, however, on separate contigs. The gap between the contigs corresponds to the likely location of the "missing" AP gene. Moreover, no unassembled reads were identified in *P. infestans *that matched PsAP3 or PrAP3 sequences.

The originally-annotated gene models for Ps_157552 and Pr_77872 both required considerable correction (Additional Files 2 and 4). The 5'-splice donor site of the third intron in Ps_157552 was identified to be a GC dinucleotide sequence. This dinucleotide has been found to be used as the 5'-donor junction at low frequency (~ 1%) in a number of fungi [27] but no information is available as yet on its use in oomycetes. The amendments to the Ps_157552 and Pr_77872 gene models (Additional Files 2 and 4) resulted in predicted polypeptide sequences that contained both signal peptide and propart segments (Table 3; Additional File 5). The sequences of the mature enzyme regions (Figure 3) showed a pairwise identity of 71%. Both polypeptides contained all three of the disulphide bonds that are common in vertebrate APs and they were only slightly extended at their C-terminal end relative to pepsin (Additional File 6). PsAP3 and PrAP3 can be predicted to display different substrate specificities. The region between residues 292 and 298 is known conventionally as the "polyproline loop" and has been demonstrated to be a major determinant for substrate specificity [22]. In PrAP3, the sequence LGDDLYW of this region has two extra (LY) residues compared to the LSDD--W sequence of PsAP3 (dashed box in Figure 3). The presence of these two hydrophobic residues is likely to influence specificity.

Like PxAP3, the PxAP4 and PxAP5 polypeptides from *Phytophthora *contained the conventional AP segments of a signal peptide and propart (Table 3; Additional File 5), prior to the mature enzyme region (Figure 3). Each trio of polypeptides was also extended at its C-terminus but, whereas the extensions in PxAP4 consisted of 136-138 residues with one putative membrane-spanning stretch, the PxAP5 extensions were substantially longer (Additional File 6). Indeed, the C-terminal extensions in PiAP5 (322 residues) and its orthologues encompassed no less than seven potential transmembrane segments (Additional File 6), an architecture highly reminiscent of the seven transmembrane stretches that constitute the super-family of G-protein coupled receptors (GPCR) [28]. Blast search using the sequence of the C-terminal extension of PiAP5 as query, identified the first non-self hit (with an E-value of 0.001) as RpkA, a G-protein coupled receptor family protein from *Dictyostelium discoideum *[29]. A 55.5% similarity in a 265 amino acid overlap was calculated between the two sequences using Smith-Waterman analysis [30]. The intriguing possibility thus arises that, in *Phytophthora *species, a fusion protein is produced in which the N-terminal partner is an AP connected to a putative GPCR as the C-terminal component. Self-processing to release the AP component would generate the mature GPCR. Such a self-processing phenomenon is unprecedented, although receptors activated by extrinsic proteinases are well-documented [31]. Further investigations into the *Phytophthora *AP-GPCR fusion-proteins would appear to be well-merited.

The final two sub-groups that were resolved within clade 3 consisted of PxAP6 and PxAP7 (Figure 1). No orthologue was detected in *P. sojae *for the proteins PiAP7 and PrAP7, encoded by PITG_05004 and Pr_73546 (Table 1; Table 2). The PiAP7- and PrAP7-encoding genes are located in co-linear orthologous regions of the *P. infestans *and *P. ramorum *genomes, as confirmed by the organization of flanking genes. In *P. sojae*, the corresponding genomic region had a similar co-linearity arrangement with the exception of two missing genes, one of them in the region where an orthologous AP-encoding gene should have been located. Moreover, no unassembled reads were identified in *P. sojae *that matched the PiAP7 or PrAP7 sequences. The simplest interpretation is that an ancestor of the PxAP7-encoding gene was present in *Phytophthora*, and deletion of the region resulted in loss of the orthologue in *P. sojae*, although it cannot be excluded that substitutions have accumulated to an extent that prevents recognition of the gene.

The PxAP6 and PxAP7 polypeptides showed an unusual variation in the component segments that constitute an archetypal AP in that a propart was absent (Table 3). In each case, the signal peptide (Additional File 5) immediately precedes the mature enzyme region (Figure 3) which, in turn, is followed by a C-terminal extension (Additional File 6). As stated above, almost all pepsin-like APs are synthesised in the form of a precursor. The propart segment makes a number of essential contributions, such as ensuring proper folding and intracellular sorting of the zymogen polypeptide, and facilitation of its activation into the mature enzyme form upon encountering the appropriate conditions [11,32]. Very recently, however, APs lacking a propart segment have been described from marine bacteria [26] and fungi [25], hence this unusual architecture of the PxAP6 and PxAP7 polypeptides in *Phytophthora *species is not without precedent.

### BACE-like Aspartic Proteinases

The PxAP8 and PxAP9 polypeptides were resolved by the phylogenetic analysis into clade 4 (Figure 1). These polypeptides also contained a signal peptide (Additional File 5), the mature enzyme region (Figure 4) and a C-terminal extension (Additional File 6) but they too lacked a propart (Table 3 and Additional File 5), just like PxAP6 and PxAP7.

The phylogenetic analysis (Figure 1) revealed that these proteins cluster within clade 4 with human BACE and its paralogue, BACE2 (sub-families A01.004/041 in the MEROPS database). The mature enzyme forms of BACE/BACE2 are initially synthesised in precursor forms that do contain propart segments. While the presence of the propart has little effect on the intrinsic proteolytic activity, its inclusion at the N-terminus of proBACE/proBACE2 ensures much more rapid folding of the polypeptide(s) than is observed when only the mature forms of each enzyme are produced [[33,34] and references therein].

BACE and BACE2 were among the first APs to be identified as having a C-terminal extension which ensured they were membrane-bound. The mature enzyme regions (Figure 4) have hallmark features which distinguish A01.004/041 subfamily members from classical APs such as pepsin. Among these are: (i) the highly-conserved Trp39 (see Figures 2 and 3) is replaced by Ala and is itself relocated to a position (residue 80) just downstream from the essential Tyr75 residue on the β-hairpin loop that overhangs the active site cleft (see Background), (ii) disulphide bonds in characteristic locations that increase stability of the polypeptide. These additional Cys residues and the Trp39Ala replacement are all present in PxAP8 and PxAP9 proteins (Figure 4).

BACE has been identified as the β-site Alzheimer's Precursor Protein (APP) Cleaving Enzyme (or β-secretase) responsible for the cleavage that generates the free N-terminal end of the Aβ peptide which plays a central role in Alzheimer's disease [35]. For almost a decade, BACE has been the target of intense efforts to develop inhibitors to alleviate the dementia of Alzheimer's disease. Genes encoding BACE and its paralogue, BACE2, have been identified in species ranging from humans through elephant, opossum, armadillo, hedgehog, shrew, and fish, to platypus (http://www.merops.ac.uk). To the best of the authors' knowledge, the identification of PxAP8-encoding genes from three *Phytophthora *species provides the first indication that BACE-like, membrane-bound APs may not be limited to the vertebrates. Intriguingly, the *Phytophthora *genomes also contain genes encoding homologues of presenilin, nicastrin, anterior pharynx defective-1(APH-1) and presenilin enhancer-2 (PEN-2) (although some gene models require substantial correction; not shown). These are all components of the human γ-secretase complex which completes the proteolytic processing by releasing the Aβ peptide at its C-terminal end from the Alzheimer's Precursor polypeptide [36]. The presence of both β- and γ-secretase gene products in microbes such as *Phytophthora *suggests that their function is not limited to the processing of a brain polypeptide related to Alzheimer's disease. These proteins therefore seem to be very ancient features that are conserved between oomycetes and vertebrates, and they have been coopted in vertebrate species to cleave the brain APP polypeptide.

While the PxAP8 polypeptides exhibited the hallmark characteristics of BACE/BACE2 subfamily members, the PxAP9 proteins lacked one feature that is necessary for an AP to be active as an enzyme. The Tyr residue located at position 75 (pig pepsin numbering) is conserved in every pepsin-like AP that is known to be active, although it does not participate directly in the catalytic mechanism [11,21]. In PxAP9, this Tyr residue is replaced by Phe (Figure 4) so the catalytic activity can be postulated to be compromised, at best. A protein engineering study revealed that the replacement of Tyr75 in a fungal AP by Asn or Phe permitted activity, albeit at a considerably reduced level, while all other replacements abolished activity [37]. However, a number of catalytically-inactive APs have been reported previously, including the pregnancy-associated glycoproteins (PAGs) found in the placenta of ungulates (subfamily A01.971 in MEROPS) [38] and perhaps most notably, the Bla g2 protein (subfamily A01.950 in MEROPS) which is a predominant allergen from cockroach [39]. The crystal structure of Bla g2 shows that it has the fold and three-dimensional structure typical of pepsin-like APs [40]. Bla g2 and the PAGs from *Ovis aries *and *Capra hircus *all have a Phe residue at position 75. These three proteins have additional substitutions at the two active site DT/SG motifs, cementing their catalytic inactivity. Their function has been postulated to include roles in peptide-binding and presentation, but without cleavage. If PxAP9 should also prove to be catalytically ineffective, the active site will still be accessible for the binding of peptides that cannot undergo cleavage and so a role similar to the PAGs/Bla g2 in peptide binding might be feasible. This intriguing possibility is rendered all the more fascinating with the realisation that, in contrast to the PAGs/Bla g2, these *Phytophthora *orthologues are likely to be membrane-bound through their C-terminal extensions (Additional File 6) and might function as a novel type of surface receptor. In this regard, it may well be noteworthy that the putative intracellular domain comprised of the residues downstream from the transmembrane segment in the C-terminal extension in PxAP9 is considerably longer than its PxAP8 counterpart (Additional File 6). Neither of the C-terminal extensions, however, contains a discernible Pfam domain or protein motif. Thus, all three *Phytophthora *species may produce one catalytically-active BACE-like enzyme (PxAP8) and one peptide-binding, membrane-bound enzymatically inactive paralogue with an intracellular extension of unknown function (PxAP9).

### Nepenthesin-like Aspartic Proteinases (MEROPS subfamily A1B)

The remaining three AP genes in each *Phytophthora *genome (Table 2), encoding polypeptides PxAP10, PxAP11 and PxAP12 cluster into clade 5 with representative APs from MEROPS subfamily A1B including the patriarch of plant origin, nepenthesin, and plasmepsin V from *Plasmodium falciparum *(Figure 1). The PxAP10 - 12 polypeptides contrast with their PxAP6 - 9 counterparts in having the archetypal AP architecture of a signal peptide followed by a propart (Additional File 5), a mature enzyme region (Figure 5), terminating with C-terminal extensions (Additional File 6). PxAP10 - 12 differed, however, from all of the other *Phytophthora *APs (PxAP1 - 9) in having a small **extra **segment inserted (dotted box in Figure 5) in the region just upstream from Tyr75 (in pig pepsin numbering). No less than seven Cys residues are present between residues 39 - 75, with a further three Cys residues (shaded in Figure 5) conserved at characteristic locations elsewhere in the mature enzyme regions of the nine *Phytophthora *APs in clade 5. Together, these Cys residues are likely to form five specific disulphide bridges, paired as indicated in Figure 5. This arrangement of disulphide bridges has been deduced from biochemical analyses of the APs of plant origin [41,42] that cluster with PxAP10 - 12 in clade 5. Indeed, most of the APs in plants [43] contain these Cys residues as well as the insert and they are grouped into subfamily A1B in the MEROPS database.

The plant APs identified in clade 5 are readily distinguished on the basis of their functional activity from one another and from others that are not included here for the sake of brevity, such as the nucellins (subfamily A01.073), regulators of the degradation of nucellar cells after ovule fertilisation [44], and the Chloroplast Nucleoids DNA-binding (CND41) proteinase (subfamily A01.050) which catalyses the degradation of RUBISCO in senescent leaves [45]. Nepenthesins (A01.040 subfamily) are extracellular enzymes secreted by the carnivorous plant, *Nepenthes*, to digest its prey [41] while CDR1 of Arabidopsis plays a crucial role in activating resistance of Arabidopsis against microbial pathogens [14]. Overexpression of CDR1 leads to constitutive disease resistance, while gene silencing compromises resistance. The CDR1 gene product is suggested to release an as yet unidentified peptide that activates a signalling cascade to induce disease resistance in a salicylate-dependent manner [14]. Recombinant CDR1 protein has been produced in *E. coli *and shown to be active as an AP in a dimeric form [42]. Two **intermolecular **disulphide bonds were proposed to be responsible for the covalent association of the two polypeptide chains in the proteolytically-active homodimer [42]. The Cys residues involved in the homodimer formation are highlighted in Figure 5 by a star (). Cys residues are present at identical locations within PxAP10 (Figure 5), suggesting that this enzyme may also function as a homodimer. In contrast, PxAP11 is devoid of cysteine residues in this region and so, based on this criterion, should function as a monomer. PxAP12 does contain two cysteine residues but at different positions within this region of the polypeptide (Figure 5). Whether these remain as free cysteines or are involved in disulphide bonds, intra- or inter-molecular, requires experimental investigation.

One feature does serve to distinguish between the plant APs and the nine PxAP10 - 12 polypeptides clustered in clade 5 (Figure 1). Most plant APs do not have C-terminal extensions (with NP_199124 from Arabidopsis included in Figure 1 as one of few exceptions). In contrast, all nine of the oomycete PxAP10 - 12 polypeptides do have C-terminal extensions, ensuring that all are membrane-bound enzymes (Additional File 6).

In addition to plant APs, plasmepsin V was resolved into clade 5 (Figure 1) as the entry most closely associated with the PxAP10 - 12 polypeptides. Plasmepsin V is one of ten APs encoded within the genome of *Plasmodium falciparum *[16], the causative agent of malaria. This enzyme (Figure 5) has a C-terminal extension with a hydrophobic stretch (not shown) that results in its anchoring as a membrane-bound polypeptide [46]. Very recently, plasmepsin V has been demonstrated to be the (aspartic) proteinase responsible for processing the precursors of *Plasmodium *effector proteins at the PEXEL motif (RxLxE/Q); this step is essential for the translocation of these polypeptides into the host red blood cell [18,19]. The malaria parasite exports hundreds of such effector proteins across the parasitophorous vacuolar membrane into the human red blood cell, thereby reprogramming the host cell to meet the needs of the parasite. Plasmepsin V is conserved in *Plasmodium *species but is not produced in vertebrates susceptible to parasitic infection and thus serves as a target enzyme against which to develop inhibitors as novel therapeutic agents for treatment of malaria.

Intriguingly, *Phytophthora *species also secrete effector proteins that are important in modulating host defence and cell death mechanisms and many such effector proteins contain the internal sequence motif RxLR, similar to the *Plasmodium *PEXEL motif [8,47,48]. *Phytophthora *effector proteins are secreted after removal of their signal peptide, giving rise to a polypeptide that contains in its N-terminal region the RxLR-containing domain which is essential for subsequent delivery into the plant cell [47-50]. It is tempting to consider that a *Phytophthora *effector precursor might undergo further processing to remove the RxLR domain, thereby releasing the mature, biologically active effector domain. The similarities described above between plasmepsin V and its PxAP10, PxAP11 and PxAP12 orthologues in *Phytophthora *suggests a conserved biological role. By analogy to the role of plasmepsin V in processing of *Plasmodium *effector proteins [18,19], one or more of its *Phytophthora *orthologues may reside in the translocon and act in the processing of *Phytophthora *effector precursors. There are obvious structural and genetic differences between the secretion mechanisms of the two parasites. The genome sequences of *Phytophthora *species lack genes homologous to the translocon complex components identified in *Plasmodium *[51]. Nevertheless, our hypothesis invites experimental investigation into targeting these particular APs in *Phytophthora *as a pathogenic Achilles heel through which the devastating losses from infection by *Phytophthora *might be reduced.

## Conclusions

One of the twelve pepsin-like APs encoded within the genomes of the three *Phytophthora *species is predicted to be produced as an unprecedented fusion protein with a putative G-protein coupled receptor as the C-terminal partner. The other APs appear to be related to existing enzymes from other species that have already been documented. These include the vacuolar enzyme, and somewhat more unexpectedly, BACE/BACE2-like APs in an active and likely inactive form akin to the Bla g2/PAG proteins, and plasmepsin V-like enzymes. Whether the latter are functional homologs of their *Plasmodium *counterpart, operating in the processing of RxLR-type effector proteins during transit to the plant cell, would appear to be worthy of immediate experimental investigation given the high level of current interest in this topic. Elucidation of the physiological roles of the APs identified here offers highly fertile areas ripe for examination. The significant revision of the erroneous gene models and the detailed annotation presented here should be highly beneficial in facilitating future experimental design.

## Methods

### Data sources and analyses

Sequence data of *Phytophthora infestans *were retrieved from the Broad Institute website (http://www.broadinstitute.org/annotation/genome/phytophthora_infestans/MultiHome.html). Sequence data of *P. sojae *and *P. ramorum *were retrieved from the website of the DOE Joint Genome Institute (http://genome.jgi-psf.org/). In cases where errors were suspected in *P. infestans *DNA sequences (gene models PITG_08190 and PITG_09837), the original trace files at http://www.ncbi.nlm.nih.gov/Traces/home/ were analyzed in more detail. In all cases the expected errors could be confirmed by analysing the trace representing the two alleles of the genes in *P. infestans *strain T30-4. The sequence error was further validated by amplification of the entire coding sequence of the genes from T30-4 genomic DNA using specific primers (underlined) linked to standard sequencing primers:

08190F1: 5'-TTTAGTGAACCGTCAGATCCGTTAAGTCAGCACGTTGTAATC-3';

08190F2: 5'-TTTAGTGAACCGTCAGATCAGTGCTGATGGACTGGCAAAC-3';

08190R1: 5'-AACAGCTCCTCGCCCTTGGGAGATGGTCAGTCCTGGTAG-3';

08190R2: 5'-AACAGCTCCTCGCCCTTGATGGTACTGTCCGACTAGTTG-3'

09387F: 5'-TTTAGTGAACCGTCAGATCATGATGCTGCGCTGCTCCGTC-3';

09387R: 5'-AACAGCTCCTCGCCCTTGCATACAGTCACCATCTTCACC-3'.

The PCR products were purified and subsequently sequenced (Macrogen, Amsterdam, The Netherlands). The corrected nucleotide sequences were deposited in NCBI (PITG_09387 = PiAP1: HM588685; PITG_08190 = PiAP8: HM588686).

Expression of the eleven *P. infestans *AP genes was determined from analysis of the data derived from the NimbleGen microarray (Accession Number GSE14480), as described previously [4].

### Identification of *Phytophthora *APs by PSI-Blast and HMMer profile alignment

A first HMMer search [24] was performed with default cut-off values of 10 using a profile based on the reference holozyme sequence set as provided by MEROPS. A second HMMer search was done with a profile that we generated based on over 5000 AP sequences identified by NCBI Entrez and verified by MEROPS batch analysis [52]. PSI-Blast was performed, using cut-off values of 10, with the PiAP1, PiAP8 and PiAP10 as queries against the NR database restricted to the taxid *Phytophthora *(which includes the complete proteome sequences of *P. infestans *but not of *P. sojae *or *P. ramorum*) using a Position Specific Substitution Matrix obtained by converging PSI-blast against the Swissprot database. PSSM generation using NR or Refseq databases failed due to high contamination in the first Blast iteration using the Blosum62 substitution matrix.

### Protein sequence analyses

Once the open reading frames of relevant gene entries were established, the resulting protein sequences were analysed in various ways. The mature enzyme region was determined by using the Pfam protein families database [53]. The SignalP server (http://www.cbs.dtu.dk/services/SignalP/) was used to predict the signal peptidase cleavage site and the absence of non-classical secretion signals in the PxAP2 sequences was verified using the SecretomeP server (http://www.cbs.dtu.dk/services/SecretomeP/). The cleavage site between propart and mature enzyme region was predicted from detailed inspection of the sequences. The prediction of transmembrane domains was performed using the web-tools ConPred II [54] or TMHMM Server (http://www.cbs.dtu.dk/services/TMHMM/) [55]. GPCR relationship was analysed by PRED-GPCR (http://athina.biol.uoa.gr/bioinformatics/PRED-GPCR/) [56].

Sequence alignments in Figures 2, 3, 4 and 5 were essentially prepared using T-Coffee [57] at the EBI server (http://www.ebi.ac.uk), and manually adjusted in a limited number of places to better align structural features that are hallmarks of APs.

### Phylogeny

The alignments shown in Figures 2, 3, 4 and 5 were combined using T-Coffee [57] with manual corrections. Non-homologous N- and C-terminal sequences were removed. A maximum likelihood (ML) tree was made using PHYML [58]. Bootstrap analysis was performed by combining the "seqboot" and "consense" commands from PHYLIP [59] and the ML algorithm from PHYML. Trees were analyzed using Dendroscope [60]. Bootstrap analysis indicated poor resolution of the PxAP2 clade. In order to improve the resolution of this clade we included the sequence of human gastricsin which was the first non-self hit detected in a Blast analysis using PiAP2 as a query. Similarly, in order to improve the resolution between Plasmepsin V and the PxAP10-12 clade, the sequence XP_002180775 from *Phaeodactylum tricornutum *(from the sister phylum of diatoms) was included in the phylogeny.

## List of Abbreviations

Aspartic proteinase (AP) genes are listed according to their identifiers (e.g. PITG, Ps or Pr) in the respective genome databases. The encoded polypeptides are identified as PiAP, PsAP or PrAP with a number. The generic descriptor PxAP with a number is used to simplify the description of any one sub-group of AP polypeptides from all three species.

BACE: β-site Alzheimer's Precursor Protein Cleaving Enzyme; PAG: Pregnancy Associated Glycoprotein; Bla g2: Cockroach Allergen from *Blatella germanica*; CDR-1: Constitutive Disease Resistance gene-1 protein product.

## Authors' contributions

JK conceived the study, performed analyses on gene models, generated alignments shown in Figures 2, 3, 4, 5 and drafted the manuscript. HJGM analysed gene models, performed co-linearity studies, reassembled trace files for gene model corrections, and sequenced genome fragments and cDNA clones to validate gene models. AtH generated the phylogenetic tree and multiple sequence alignments. JALvK analysed gene models, coordinated the entire study, and prepared the final manuscript. All authors contributed to writing the manuscript and approved the content. They declare that they have no competing financial interests.

## Supplementary Material

Additional file 1**Amino acid sequence of pig pepsin with characteristic motifs and features of Aspartic Proteinases**. The DTG and hydrophobic-hydrophobic-Gly motifs forming the psi loops in each domain are boxed, as are the landmark residues Tyr75 and Trp39. The Cys residues contributing the three disulphide bonds are shaded and their pairing is indicated by thick () black lines.Click here for file

Additional file 2 **Corrections made to erroneous *Phytophthora *Aspartic Proteinase gene models (as represented in databases in January 2010)**. Click here for file

Additional file 3 **Gene expression analysis of *P. infestans *AP genes by NimbleGen microarray**. Samples used in the hybridization were isolated from *P. infestans *grown *in vitro *on three different agar types (pea agar, V8 juice agar rye sucrose agar) or from *P. infestans*-inoculated potato leaves at 2, 3, 4 and 5 days post inoculation. Details of the experimental procedures of the microarray analysis are provided in [4]. Values +/- SD are the averages from two independent experiments. Click here for file

Additional file 4 **Corrected *Phytophthora *Aspartic Proteinase gene models**. Predicted protein sequences of *Phytophthora *Aspartic Proteinases PxAP1-12. Bold characters represent the predicted signal peptide. Underlined characters represent the predicted propart segment. Italic characters represent the C-terminal extension. Non-bold, non-underlined, non-italic characters depict the mature enzyme region. Amino acids marked in yellow are the residues added to the protein sequence as a result of the revision of the gene models listed in Table 1. For each entry, the database accession number for the gene is given, followed by our allocated identifier (in parentheses) for the encoded polypeptide. The corrected gene models for *P. infestans *genes PiAP2, PiAP4, PiAP6, PiAP7, PiAP9, PiAP11 and PiAP12 have been implemented in the Broad Institute database (October 2010). Gene models for PiAP1 and PiAP8 in the Broad Institute database were not corrected because these genes contain sequencing errors. The correct gene models for PiAP1 and PiAP8 can be retrieved from NCBI accession number HM588685 and HM588686. The corrected gene models for *P. sojae *genes PsAP3, PsAP9, PsAP11 and for *P. ramorum *genes PrAP3, PrAP8, PrAP10 and PrAP11 have not yet been implemented in the JGI databases (Apil 2011). Click here for file

Additional file 5 **Signal peptides and propart segments at the N-terminal end of the mature enzyme regions of PxAP1-12**. Gaps were introduced to optimize alignment. Each signal peptide and propart segment is spatially separated and marked by the respective boxes at the top. The lack of a signal peptide or propart segment in individual polypeptides is indicated by ------ none -------. Click here for file

Additional file 6 **Alignment in orthologous sets of the extensions at the C-terminal ends of the mature enzyme regions of PxAP2 - 12 detailed in Figures **2, 3, 4, 5. Gaps were introduced to optimize alignment. Hydrophobic residues contributing potential membrane-spanning stretches are boxed. Click here for file
