# Supplementary material for: The aspartic proteinase family of three Phytophthora species
Source: BMC Genomics. 2011 May 20;12:254. doi: 10.1186/1471-2164-12-254 (PMC3116508; doi:10.1186/1471-2164-12-254)
Supplement: Additional file 1 — Amino acid sequence of pig pepsin with characteristic motifs and features of Aspartic Proteinases. The DTG and hydrophobic-hydrophobic-Gly motifs forming the psi loops in each domain are boxed, as are the landmark residues Tyr75 and Trp39. The Cys residues contributing the three disulphide bonds are shaded and their pairing is indicated by thick () black lines. [file 1471-2164-12-254-S1.PDF]

Amino acid sequence of pig pepsin with characteristic motifs of Aspartic Proteinases.

```
IGDEPLENYLDTEYFGTIGIGTPAQDFTVIFDTGSSNLWVPSVYCSSLACSDHNQFNPDDSSTFEATSQELSITYGTGSMTGILGYDTVQVGGISDTNQI
1                  20          32            45   50                             75             90

FGLSETEPGSFLYYAPFDGILGLAYPSISASGATPVFDNLWDQGLVSQDLFSVYLSSNDDSGSVVLLGGIDSSYYTGSLNWVPVSVEGYWQITLDSITMD
        110         122              140                 160                  180                200

GETIACSGGCQAIVDTGTSLLTGPTSAIANIQSDIGASENSDGEMVISCSSIDSLPDIVFTINGVQYPLSPSAYILQDDDSCTSGFEGMDVPTSS
             215            230                249                    270         282

GELWILGDVFIRQYYTVFDRANNKVGLAPVA
   300
```
