# Supplementary material for: The aspartic proteinase family of three Phytophthora species
Source: BMC Genomics. 2011 May 20;12:254. doi: 10.1186/1471-2164-12-254 (PMC3116508; doi:10.1186/1471-2164-12-254)
Supplement: Additional file 2 — Corrections made to erroneous Phytophthora Aspartic Proteinase gene models (as represented in databases in January 2010). [file 1471-2164-12-254-S2.PDF]

Corrections made to erroneous *Phytophthora* Aspartic Proteinase gene models.

Species	Gene model number	Type of correction	Corrected protein length	Corrected #introns
P.infestans	PITG_09387	Sequence error, insert 1 nucleotide, frame shift, no intron.	390	0
	PITG_12357	Start codon moved upstream, adding 3 residues.	472	0
	PITG_05114	Second intron rejected by EST, in frame insertion of 14 amino acids.	559	1
	PITG_04975	Start codon moved upstream, adding 36 residues; intron incorrectly predicted leading to insertion of 19 residues.	566	0
	PITG_05004	First two exons wrongly predicted; moved the start codon downstream by approx. 800 nt.; intron was incorrectly predicted.	444	0
	PITG_08190	Sequence error, insert 2 nucleotides, frame shift, no intron, leading to insertion of 22 residues.	574	0
	PITG_06900	Inserted one exon in front of predicted gene based on ESTs, leading to insertion of 40 residues and removal of 8 residues at the N-terminus; two introns incorrectly predicted, leading to insertion of 132 and 10 residues in the C-terminal region.	647	4
	PITG_02624	Inserted two exons in front of predicted gene based on ESTs, leading to insertion of 181 residues; moved 3’-splice junction of last intron upstream by 321 nt, thereby inserting 107 residues.	638	4
	PITG_11522	Start codon moved upstream, adding 32 residues; first intron incorrectly predicted, leading to in frame insertion of 18 residues; second intron incorrectly predicted, leading to in frame insertion of 26 residues.	522	0
P. sojae	Ps_157552	Both the 5’- and 3’- splice junction of intron 3 were adjusted; intron 5 was incorrectly predicted, leading to frame shift and stop codon 33 residues downstream of predicted exon 5.	425	4
	Ps_135764	Inserted one exon inside the first intron, and adjusted the 3’-splice junction of the resulting second intron, thereby inserting 27 residues.	654	4
	Ps_158877	First two exons deleted; start codon in third exon moved downstream; one 3’-intron junction incorrect; two introns wrongly predicted, sequence continues in frame; insertion of 18, 28 and 31 residues, respectively; last three introns non-existent.	642	4
P. ramorum	Pr_77872	Moved the 3’-splice junction of intron 1 upstream, thereby inserting 52 residues; moved 3’-splice junction of intron 4 downstream, leading to frameshift and stop codon 51 residues downstream.	414	4
	Pr_78678	Start codon moved downstream, deleting 25 residues.	586	0
	Pr_77495	Moved the 3’-splice junction of intron 1 upstream, thereby inserting 14 residues.	650	2
	Pr_77493	Moved the 3’-splice junction of intron 3 downstream, leading to extension of exon 3; added two more exons, leading to addition of 285 residues to the C-terminal end.	640	4
