# Supplementary figures and images for: The aspartic proteinase family of three Phytophthora species
Source: BMC Genomics. 2011 May 20;12:254. doi: 10.1186/1471-2164-12-254 (PMC3116508; doi:10.1186/1471-2164-12-254)

**Gene expression analysis of *P. infestans* AP genes by NimbleGen microarrays.**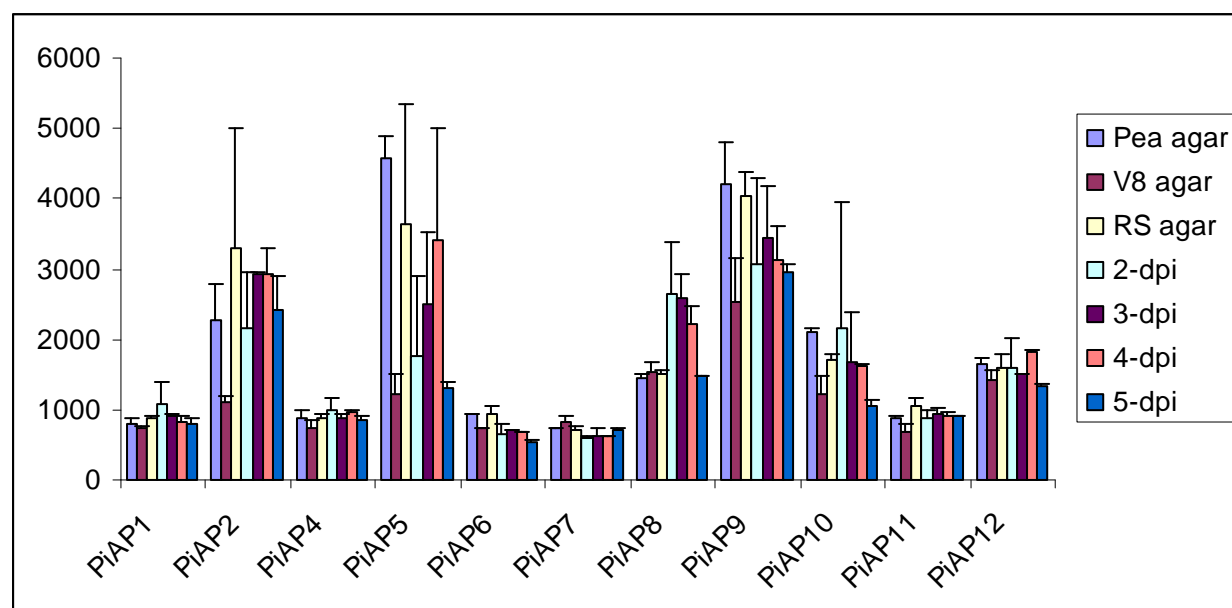

Supplement: Additional file 3 — Gene expression analysis of P. infestans AP genes by NimbleGen microarray. Samples used in the hybridization were isolated from P. infestans grown in vitro on three different agar types (pea agar, V8 juice agar rye sucrose agar) or from P. infestans-inoculated potato leaves at 2, 3, 4 and 5 days post inoculation. Details of the experimental procedures of the microarray analysis are provided in [4]. Values +/- SD are the averages from two independent experiments. [file 1471-2164-12-254-S3.PDF]
