# Supplementary material for: The aspartic proteinase family of three Phytophthora species
Source: BMC Genomics. 2011 May 20;12:254. doi: 10.1186/1471-2164-12-254 (PMC3116508; doi:10.1186/1471-2164-12-254)
Supplement: Additional file 4 — Corrected Phytophthora Aspartic Proteinase gene models. Predicted protein sequences of Phytophthora Aspartic Proteinases PxAP1-12. Bold characters represent the predicted signal peptide. Underlined characters represent the predicted propart segment. Italic characters represent the C-terminal extension. Non-bold, non-underlined, non-italic characters depict the mature enzyme region. Amino acids marked in yellow are the residues added to the protein sequence as a result of the revision of the gene models listed in Table 1. For each entry, the database accession number for the gene is given, followed by our allocated identifier (in parentheses) for the encoded polypeptide. The corrected gene models for P. infestans genes PiAP2, PiAP4, PiAP6, PiAP7, PiAP9, PiAP11 and PiAP12 have been implemented in the Broad Institute database (October 2010). Gene models for PiAP1 and PiAP8 in the Broad Institute database were not corrected because these genes contain sequencing errors. The correct gene models for PiAP1 and PiAP8 can be retrieved from NCBI accession number HM588685 and HM588686. The corrected gene models for P. sojae genes PsAP3, PsAP9, PsAP11 and for P. ramorum genes PrAP3, PrAP8, PrAP10 and PrAP11 have not yet been implemented in the JGI databases (Apil 2011). [file 1471-2164-12-254-S4.PDF]

Corrected *Phytophthora* Aspartic Proteinase gene models.

Predicted protein sequences of *Phytophthora* Aspartic Proteinases PxAP1-12. Gene models that required adjustment are listed in Additional File 1. Bold characters represent the predicted signal peptide. Underlined characters represent the predicted propart segment. Italic characters represent the C-terminal extension. Non-bold, non-underlined, non-italic characters depict the mature enzyme region. Amino acids marked in yellow are the residues added to the protein sequence as a result of the revision of the gene models listed in Table 1. For each entry, the database accession number for the gene is given, followed by our allocated identifier (in parentheses) for the encoded polypeptide. The corrected gene models for *P. infestans* genes PiAP2, PiAP4, PiAP6, PiAP7, PiAP9, PiAP11 and PiAP12 have been implemented in the Broad Institute database (October 2010). Gene models for PiAP1 and PiAP8 in the Broad Institute database were not corrected because these genes contain sequencing errors. The correct gene models for PiAP1 and PiAP8 can be retrieved from NCBI accession number HM588685 and HM588686. The corrected gene models for *P.sojae* genes PsAP3, PsAP9, PsAP11 and for *P. ramorum* genes PrAP3, PrAP8, PrAP10 and PrAP11 have not yet been implemented in the JGI databases (April 2011).

>PITG_09387 (=PiAP1; Genbank accession number HM588685)
MMLRCSVLCSLLLALSSAAVMRVPMIKRSDDEFVSSLLHDVHAMQRPVVWSPDASPINNQNEGQSTVEGSASVVIRDFQNAQYY
GEISIGTPPQPFAVIFDTGSSNLWVPDKKFGSHNVYDHDKSSTYKPNGTAFDIMYGSGPVSGFLSQDKLELGGLTVPDQYFAEV
NVTKGLGPAYYLGKFDGLFGLAFDTISVDHLKTPFHRMVQEGLLDEPVFAFYLGDQKDGELTFGGVDKAHYKGEIEYVDVTSAT
YWSVKLGAVETKGEKLTDVDKAIVDSGTSLIAGPKDQVAKLAALVGAHKFIMGEYLISCTAAAPDISFVLNGKTYTLTKEEYTL
KSGPICLFAFMGIDIPAPAGPLWILGDVFMRKHYTVFDWGTDSRKPRVGFALAA

>Ps_109366 (=PsAP1)
MLRCSLLCSALLALALCASDAAVLRVPMRKRSDDEFVASLLHDVHALNRPVVWAPEADAFPSPATDADAVQGTANVVIRDFQNA
QYYGAISIGTPPQPFAVIFDTGSSNLWVPDKKFGSHNVYDHDASSTYKANGTAFDIMYGSGPVSGFLSQDTLALGGLTVPDQFF
AEVNVTKGLGPAYYLGKFDGLFGLAFDTISVDHLKTPFHRMIQQGLLDEPVFAFYLGDQKDGELTFGGTDKAHYKGELEYVDVT
SATYWSVKLDAVEAKDQQLTDVNKAIVDSGTSLIAGPKDQVAKLAALVGAHKFIMGEYLISCTAAAPDISFVLNGKKYTLTKDE
YTLKSGPICLFAFMGIDIPAPAGPLWILGDVFMRKHYTVFDWGTDSRKPRVGFALAA

>Pr_77239 (=PrAP1)
MLRCSLLVSALLLASDAAVLRVPMIKRSDDEFVSSLLRDVHALQRPAVWSPQEPNSDTFNPDVENVEDSANVVIRDFQNAQYYG
AISIGSPPQPFSVIFDTGSSNLWVPNKKFGAHKVYDHDASHTYKANGTEFKIQYGSGPVSGFLSQDELQLGGLTVADQFFAEVN
VTKGLGPAYYLGKFDGLFGLAFDTISVDHLKTPSHRMIQQGLLDDPVFAFYLGDQKDGELTFGGVDKAHYKGELEYVDVTSATY
WSVALDAVQTKGEKLTDVHKAIVDSGTSLIAGPKDQVAKLAKLVGAYKFIMGEYLISCTAAAPDISFVLNGKTYTLTKDEYTLK
SGPLCLFAFMGIDIPAPAGPLWILGDVFMRKHYTVFDWGTDTRKPRIGFALAA

>PITG_12357 (=PiAP2)
MTSMAVAKVPMRRGQTHWTRSRRLVSSASNSNAVDLENYQGTIYFADIVVDGSNFVVQVDTGSSDLWISCYYLSGSTCQSTCPS
DAETISYGSGDVCVEGESADIKFGNVAVSNYNVGVAQGSTVNPSSSSSLLAGDAQGLFGLAYSSLATLPSPGGQFIDYVQSFSM
YLTQEDNSAGSFLLLNGVDDALISSNGLTPYTVKLKSATPTHWTIGMTAMQIGNSTAVFPCSTASSGSCDSIVDSGTSLLAMPS
SVYSSFVSTYLSSCQRDQSGSEIYMCSSDIELPRLALTFGDVTFYLEKNDYIINLGGNMVVVELQATSSASGSYANTWIIGETF
LKIFYTSYNVNESVTFYCVENSTC*TPGSQAVSLPASSDSSGDNTGGNGFGGGSGGISGITFNDDDKSHLTTILAIVLGVLGFFL*
IIAVVTSLFRCMCRRRRASRHEQSLQPVLVVPGGYYQPQPAIQPSQGYYAQH

>Ps_144332 (=PsAP2)
MTTSKAVAKVPMRRGQSPWTRSRRLASSASNSNAVDMENYQGTIYFADIVVDGSTFVVQVDTGSSDLWISCYYLSGSTCSSTCP
SDASTISYGSGDVCVEGTSADIKFGNVAVSDYVVGVAQGSNVNPSSSSSLLAGDAEGLFGLAYGSLATLPSPGGQFIDYVTSFS
MYLTKEDNSDGSFLLLNGVDDELISSNGLMPYTVDLKSSTPTHWTIGMTAMQIGNNSAVFPCSTASSGSCDSIVDSGTSLLAMP
SSVYSDFVSTYLSGCQQYSSGSEIYMCSSDVDLPRLALTFGDVTFYLEKDDYIIDLGANMVVVEVQATSSSSGSYANTWIIGET
FLKTFYTSYNVNESVTFYCVENSTC*TPGSQAVSLPASSDSSGDNTGGGGFGSGSDGTSGTLSSDDKSHLVTILAIVLGVLAFFF*
IIAVVTYLFRCICRRKRASRHEQSLQPVLVVPGGYYQPQPAVQPSQGYYAQH

>Pr_84847 (=PrAP2)
MASKVPMRRAQSPWTRSRRLVSSDSNSNAVDMENYQGTIYFADIEMDGSSFVVQVDTGSSDLWISCYYLSGSTCESSCPSDAST
ISYGSGDVCVEGTSADIRFGNVAVANYVVGVAQGSTVNPSSSSSLLAGDAEGLFGLAYSSLATLPSPSGQFIDYVTSFSMYLTQ
EDNADGSFLLLNGVDDALISSGGLTPYTVNLKSSTPTHWTIGMTALQIGNNSAVFPCSTASSGSCDSIVDSGTSLLAMPSSVYS
DFVSTYLSGCQEYSSGSEIYMCSSDVELPRLALTFGDVTFYLEKEDYIIDLGGNMVVVEVQATSSSSSSYANTWIVGETFLKVF
YTGYNVNESVTFYCVENSTC*TPGSQAVSLPASSDSSGDNTGGNGFGGNSDGGTSGTSSSDDDKSHLVTILAIVLGVLAFFFIIA*
VVQYLFRCVCRRKRASRHEQSLQPVMVVPGGYYQPQPAIQPSQGYYAQH

>Ps_157552 (=PsAP3)
MIHRWVVLLACAVAVVCIAPCSAMIRIPLSRRQRGGIELSEPAAAMQGLSTVTHTHSKLSHLPLRNFENDQYFGFVAVGTPPQT
FRVLFDTGSSDAWLPDQSCSTCGSHTRFKRHRSRSFRATTDTFWGIYGSGDSYGLVGADVFTIGNYSVPDLAFAVITEETGGIS
ALANDGVIGLAFAGMSKISRPTILDTIKSSNPELNLQFAFYIAGAAQKDDSEFHFGGYDLSVAGERAALAKFPVLTLPIDPQLT
YWTLAVNDFHLVHTAGIRKSSNLCDPFCYAIIDSGTSFIYVPPQLYDSVITEVVAGKACDLEQLTCANTGYESFPTLSFSFGSI
HDGNFFHLGPRSYLNCYQDTCDIELLNHASLSDDWWVLGGRFLREYYTVFDFDSMQVGIACS*GWSSHCRARLQLEEKSHVLLFV*
SNVNS

>Pr_77872 (=PrAP3)
MQWCECAALLVYLLIAVCAARCSAVVRIPLSMRRRGISPQAAAMQELSTPTHSVSIHAKISQVPLHNFENDQYVGLVTVGTPPQ
SFRVLFDTGSSDAWLPDQSCSTCGDHARFVRHQSSSFQPTTETFRGIYGSGDSYGLVGIDGFTIGNYSVPELAFAVLTEETGDI
PALANDGVIGLAFAGMSKVAHPTILDIVSNSNPDLMPVFAFHLTDETEEEASEFHFGGYDLSVAGEGAALAKFPILTLPTATEP
TYWTLAVSDFHVVRQGKSSNLCEPLCYAILDTGTSFIYVPPQLYDSVIAEVTAGKSCDLDQLICENVGYESFPTLSFSFGSSND
GNFFHLGPQSYLDCGQDSCYIELLNHASLGDDLYWWVLGDNFLHEYYTVFDFQKLQVGIACG*GSSPRCSIGEYKMPST*

>PITG_05114 (=PiAP4)
MRLRLLPSLAILSTHCVATGAEPLRVSLARRRPPTAATPDWIQSEAVAYHHGDPATPDWIMTESNSTQDAAQPPLPHGKRVPLQ
NFGNVQYIGAVGFGNPPQYFDVVFDTGSSDTWIPGTSCNSCGSHHQFDSQKSTTFLDTEEKFYDAYGSGSVSGTVVVDTVTISG
YTVDSVRFGIIDDESDKLQEFLADGIFGLGFEGLAHISRPTVFAALAGQNADLENMFAFYLTPEAYRTGSELHIGGYDLSVVGP
NASFHYTPVVKLPEFDSFMYWTIKMNNFSIVPQTLSDGDSINAEVTVQADVSVNMCEPFCYAIVDTGTSLISVPAGQFNEVVSK
ITRGLDCDGIDCNAVAVKDFPVLHFGMEPDNVFLLQPQDYVLCSGWGQCKLQFQSTTDEWWILGDVFIKTYYTLFDAERMRIGF
ACD*GDVCQGGRGNIYGDNGEGGAFGAWENAFLLGSCFAAACMFLFVFLLNQQDEEPNLSQDEGFLGAKIDRRNSPQGFQTHDPK*
RPLLYDDEESQHVYATSSSSTSQASSYAEARRNFAALPSPRSSDGYQSDGSCVEV

>Ps_132274 (=PsAP4)
MRLRLLSLLTLLAPSQCAASDAGPLRVSLARRRSPPDTAPAVASATTATATDWIQSEARVYHTHAPVPEWTVAEANATQDAAQP
PLPTAKRVTLQNFGNVQYIGTVGFGNPPQYLDVVFDTGSSDTWIPGTSCDSCGSHHRFDSQRSTTFLDTEEKFYDAYGSGSVSG
NVVVDTVTISGYTVDSVRFGVIDDESDKLQEFLADGIFGLGFEGLAHISRPTVFAALAGENADLENMFAFYLTPEAYRAGSELH
IGGYDLSVVGPNASFHYTPVVKLPEFDSFMYWTIKMNDFSVLPPVGKSSKSSDANNGDNMESDVAANMCDPFCYAIVDTGTSLI
SVPAGQFDEVVSKITRGLDCVGIDCDAADVKDFPVLHFGMEPDNIFLLQPQDYVLCSGWGQCKLQIQSTTDEWWILGDVFIKTY
YTLFDAERMRVGFACE*GDICQGGRGNIYGDDGEGGAFGAWENAFLLGSCFAAACMFLFVFLLNQQDEEPYLNQDESFLGGIIDR*
RTNSPQGYQVHDPKRPLLYDDEDQHAYATSSTSTSQASSYAEARRSFAALPSPPGSTDGHQSDGSCVEV

>Pr_82845 (=PrAP4)
MWLHLLTALALLTSQFAHAAPFKLSLARRLPATDWIQSEANAYRAHPAAPEWTVAEANATQDAAQPPLPSAKRVTLQNFGNVQY
IGTVGFGNPPQLMDVVFDTGSSDTWIPSSSCESCGSHHQYDAQKSTTFLDTEEKFYDAYGSGSVSGTVVVDTVTISGYTVDSVR
FGVIDDESDKLQEFLADGIFGLGFEGLAHISRPTVFAALAGQNAELENMFAFYLTPEAYRTGSELHVGGYDLSVVGPNASFHYT
PVVKLPEFDSFMYWTIKMNNFSIQPVGDDNNNNGETDVTANMCDPFCYAIVDTGTSLISVPAGQYDEVVSKITRGLDCDGIDCD
GVAVKDFPVLHFGMEPDNVFLLQPQDYVQCSGWGQCKLQFQSTTDEWWILGDVFIKTYYTLFDAERMRVGFACE*GDICQGGRGN*
IYGDNGEGGAFGAWENAFLLGSCFAAACMFLFVFLLNQQDEEPNLNQDEGFLGALIDRRANSPSGYQTHDPKRPLLFDDEESQH
VYATSSTTTSQASSYTEARRSFAALPSPPGSNDGHMSDGSCVEV

>PITG_10524 (=PiAP5)
MRLGLLVAALAALSFQPASSLLRVPLTAKAQRRSADHLLQFHTQPSDAVTNAVQADHVLQYAAINTQDILHNAQDLAAQGLVPL
ENFMEFQFFGPIAIGSPPQEVLVCFDTGSSDLWVPGNKCEACAGQDRFNHSLSSTYHESTTHPAFAVQYGSGKVSGHFGLDVVH
VAQFQVQDTTVGIVRTEEESMARMKADGLLGMAFDGLSTFSHPPLFFALLEQYPELDSVFAFYLSPDPNTNGSELHLGGYDEDF
MGSLEASWQMTDVLPQFGQWTFWRIHLHSVNVGKHRNACADGCVAFVDSGTSLIGIPGTLYLNFLYEVATFAQNQGCYCGFVQY
GFQCFLCAPEDFPPLRIGIGGNHFYVLEGSDYTLCVGLTCIVLVQPSGQEMWVLGDVFMKKFYSLYDVKKKQMGFACPA*NSTLC*
GVEDMQDKTVNSNGNDLLPSQKSPFFENSFNMYDMDTHAVLVLFLSGLSLVGSGFIVSSFVQYPILRSFRSFSIFFWLSVCTLG
YNLTLWIAGVWRAHQTHVLFCALLKSSQQFFGTAILLFSAAVGLELIRAVRWTHSSTVEYKLVYHVVIWCSAAFTGAFSLLTGV
VGFLPDGAGPCRACFVGHSPGWARVLLFYLPATLTLTFAGSAVYLASKGSAGLSLPPQAERARRSSGQLLSSCVATVAALFLPT
LFGWLQSFGADWVTSGFFLYLSELCFYSQGLLNALSWAFNPSYRVARYRGSNAVGGEATRLMGPN

>Ps_138576 (=PsAP5)
MAATPGRRRLALALALLSLLSLTLQRVHSVAAPLRVPLRAASKRRNAQKLLQFHTQPEPKAAAALNVDGVVSSLRARQPEAAGL
HDAQQLAAEGHVPLENFMEFQFFGPVQIGTPPQEVLVCFDTGSSDLWVPGRSCEACAGLDRFNHSQSSTYAESSTRPAFAVQYG
SGKVSGHFGQDVVRVAQFEVRDTTVGIVHTEEESMARMKADGLLGLAFDGLSTFSHPPLFFALLEQYPELDSVFAFYLTPDPNS
NGSELHLGGYDQEFMDSLQATWQMTDVLPQFGQWTFWRIHLHSVNVGKHRNACADGCVAFVDSGTSLIGIPGTLYLNFLYEVAT
FAQNQGCYCGFVQYGFQCFLCAPEDFPPLRIGIGGHHYYVLEGSDYTLCVGLTCIVLVQPSGQEMWVLGDVFMKKFYSLYDVKK
KQMGFACPT*NSTLCGVEDIDETVSSDDTDGLPSKVSSPFFENSFNMYDMDTHAVLVLFLSGLSLVGSGFIVSSFVQYPILRSFR*
SFSLFFWLSVCTLGYNLTLWIAGVWRAHQTHVFFCALLKSTQQFFGTAILLFSAAVGLELIRAVRWTHSSTVEYKLVYHVVIWC
SAAFTGAFSLLTGVIGFLPDGAGPCRACFVGHSPGWARVFLFYLPATLTLTFAATAVYLAYTSNAGQSLPPQAERARRSSGQLL
SSCVATAAALFLPTFFGWLQAFGAEWVTTGFLLYLSELCFYSQGLLNALSWAFNPSYRVARYRGNNATGGEGMRLMGPN

>Pr_84399 (=PrAP5)
MLNRLRLAALLAAFVTLALPSQGANSLLRIPLTATTQRCSAQKLLQFHTQPSDAVTTNAVEAEGVLSSLNVLQNGDNSLRAQDT
LHNAQQLEAEGHVPLENFMEFQFFGPIAIGSPPQEVLVCFDTGSSDLWVPGKKCEACAGLDRFNHSQSSTYHESTTHPAFAVQY
GSGKVSGHFGQDVVHVAQFEVQDTTVGIVRTEEESMARMKADGLLGLAFDGLSTFSHPPLFFALLEQYPELDSVFAFYLSPDPN
TNGSELHLGGYDEDFMGFMQASWQMTDVLPQFGQWTFWRIHLHSVNVGKHRNACADGCVAFVDSGTSLIGIPGTLYLNFLYEVA
TFAQNQGCYCGFVQYGFQCFLCAPEDFPPLRIGVGGHHYYVLEGSDYTLCVGLTCIVLVQPSGQEMWVLGDVFMKKFYSLYDVK

KKQMGFACPA*NSTLCGVEDFEKAAGDSNDNDLLPSKQASPFFENSFNMYDMDTHAVLVLFLSGLSLVGSTFIVSSFVQYPILRG*
FRSFSLFFWLSVCTLGYNLTLWIAGVWRAHQTHVFFCALLKSTQQFFGTAILLFSAAVALELIRAVRWTHSSTVEYKFVYHVVI
WCSAACTGAFSLLTGVIGFLPDGAGPCRACFVGHSPGWARVFLFYLPATLTLTFAGTAVYLAYTGSSGQSLPPQADRARRSSGQ
LLSSCVATVAALLLPTLFGWLQAFGTDWVTSGFLLYLSELCFYSQGLLNALSWAFNPSYRVARYRGANATGGEAMRLMGPN

>PITG_04975 (=PiAP6)
MGPRRVRLAAMALSLFCPCANSTTELIRIPLENYDQMQFFGSIGVGSPPQRFQVIFDTGSSDIWLPESSCADCAGSRRYHAAVS
RSHEPLNEPFRLEYGSGNASGRVVREQVSLFGGDEQSLTLSRVHMGSTTKTTKSLQRFQADGIVGLGLEALAVITKPSLLKSDP
RLGLFSIYINPLPGALPPAQLIFGGVDDSLPIAHIPHANEAQVSWHHFPLVRYPSSRRAHGFWAIRLHRLTVGNFDSRSKSSPK
KLAGSGDGIVAVSAAVAIVDSGTSLLLLPRRAFDTTITEIQQHLRVQHSKELRVNPHAVSGYACVDCTAEMFPALRFSFVIEET
PAGSQSKTQTLVLKGTDYARCDDLMCAPQLDVHALFTSKSKSKVPTPAASAMTTNIPQGTEHEDVVVLGVTFMRAYYVQFDSQR
KTVGFACV*DSATSSAVCSGGWAPKLQFHSAHFLDAEASAWRAGWVFWSRVYLGIGVLLLLVAFALLWLILVVPSSEVKKVFNWF*
YGPSDKSRRRQQQTECKLLPTRRSSYQGQSSDSNRDDETAEPDSEPESPPAPSRRKSVSFDV

>Ps_136075 (=PsAP6)
MVPHRRLRHAAAGLALTLMGARAAAAPAELLRIPLENFDQMQFFGSIGVGSPPQRFQVIFDTGSSDIWLPETNCADCAGSRRYS
AAVSLSHEALDEPFRLEYGSGNASGRVVREQISLFGGDAETDVLTLSGVHIGSASKTTKRLQRFQADGIVGLGLEALALITKPS
LLQSEPRLRRFSIYINPLPGALPPAQLIFGGVDDTLPVAHAPLESGAKVAWHHFPVVRYPSSRRAHGFWAIRLNRLSVGSFDSR
SDSSSKQLAGSGEGIVAVSAAVAIVDSGTSLLLLPRRAFDATIAEIQQHLRVQHRRELRANPHAVSGYACADCTAEMFPALRFS
FALEEASAGGMPPKTQTLVLQGTDYARCDDFICAPQLDVHALFTSKKNKSKQQTPVASAMTTNIAQGKEYEEVVVLGVTFLRAY
YVQFDSELKTVGFACV*ESSASSSSGGANVCSGGWAPKLQFHSTHFSDAEASTWRAGWVFWSRVYLGVGVLLLVVAFALLWLILV*
VPSSEVEKVLDWFFGPSERSRRRQQQQVSTDSKVASSARGSYQTLENGVPIGGGDGEEAPVEPDAEPESPRSASRRRSVCYDV

>Pr_73566 (=PrAP6)
MARRRLRCAAVALSLAACASSPSAAELLRIPLENYDQMQFFGSIGVGSPPQRFQVIFDTGSSDIWLPETKCADCAGSRRYHAAV
SRSHEALDEPFRLEYGSGNASGSIVREQISLFGGDAQQDTLTLAGVHMGSASKTTKRLQRFQADGIVGLGLEALALITKPSLLK
SDPRLQRFSFYINPLPGALPPAQLIFGGVDDSLPVAHLPHANQSQVAWHHFPVVRYPSSRRAHGFWAIRLHRLTVGSFKSRSDT
GSKQLSGSGEGIVAVSAAVAIVDSGTSLLLLPRRAFDATIAEIQQHIRVEHDRELRANPHVVSGYACADCTPEMFPALRFSFVM
EEASGGSPPKTQTLVLQGTDYARCDDLICAPQLDVHALFTSKKSKQVHTPATSAMTTNVPQGKEHEEVVVLGVTFLRAYYVQFD
SARKMVGFACV*EPSLLSGESGVCSGGWAPKLQFHSTHFSDAEASAWRAGWVFWSRVYLGVGVLLLLVAFALLWLILVVPSSEVE*
KVLDWLCGPSSKSRRRQQQPPLDSKASSGCHGTPQALESGISSDNRDCSNAHEASEEPDSEPAAATAPSRRKSVCYDV

>PITG_05004 (=PiAP7)
MDRCKRILRYLIVLVLLLTQDFSAASQCHFIRVPLDSDDQLRFTGVVRLGTPPRSVRVIFDTGSSDTWVSSNYAADVGARSASR
AFTLGYGGGIVSGLVAHTDLQLRSNPADVILLPVGFVNDHTSVLSGLDAQGVVGLGMEALAQIQSNWSLVGLLAQQSRMKPLAV
SLFISSWSQAQPASLLIIGGVDPALLTANATWFSFPVVSNIQSSKTGYGFWALTLQNLSFGNESLPFGGPSRSSGIALLDSGTS
VLLLPRHTFDTVIHVLSVRFGTRLLSPPNKKPHIPVCRPCHVHEFPSLAFEFVEFNAMIRRFELHGSDYVRCDHRRRECTAMID
VIDSSEVSDHLDVVILGTVFFRAYYTRFDYSNKQISFACT*SDDNVCLGGLQPALDYHGKPYEQYSSVQQPCQLWAGLCAVATAL*
AVFAGLRTLLKTLHADPRIYINNG

>Pr_73546 (=PrAP7)
MARQLYCWLVLVHAIAATSLVRIPLESEDHLSFTGAVHLGSPPQRARVVFDTGSSDAWVTSAFAFAVSGLSAGETITIGYGGGL
VAGLAGWTDLRLLGSDENELLLHDVHVGCVDDQTSELPGVDAQGVFGLGMETLAQIHSNSSFLGTLASPVAFSLYISSSSGAQP
SSQLSFGGDDPALTSPSTKWFTFPVASNDALRGAHPSSSSTKGTFGFWALPVQSVSFDKILLPSGGPDKHHIIALFDSGTSMLL
LPPRVFDSVVQALVVRFGTRFLPPTKRQSLPACRLCRVNEFPPLAVEFLLDDGHSPDAKSQRFVLQSSDYVRCDQHRHECTAMI
DFIHPSELSSDVDVVVLGTVFFRAYYAHFDYTHKQVGLACT*VDDNGVCPGGVQPALDYRGRPYESFEDRRQSSPSFVGLCAAAT*
ALALLAGLQMVL

>PITG_08190 (=PiAP8; Genbank accession number HM588686)
MRIAVATALLTQTAQASNVLQQQLWGLSSGLAYYVEVNIGSPLYSSGSSSSSSNSFNLLLDTGSANTAVVTAECCSLTNQNLYS
CSDSSTCVDEGASVSVSYVSGAWSGEVVRDTFSGQGLGTVENMQFAEITAEDSFISSGYDGIIGLGYKSIASPSSNPPTPYFDT
VLSADGLANVFSLQMCGALQALSLSNVSTEDGSHLYAGEFLLGGTEGPNGESYHKGDIVYTPLVQEKYFNVIITDIGVNGESLG
LDCESINSPRSIVDSGTSNIAFPSSVYSAIIAELKTQVERIATVSDSFFDDDTTCCSSDCDPNNADSIIYQLPGLTISLAVDGD
NSQQMTITIPAEYIWRPIVVSTGRGEAACRVFGISEGDFTLLGDVFMDGLFTVHDRANERVGLAIADNCPNGVTSSKNITIETM
TAEDSLC*DCVGSADRKSSLISSYVPFSGKPCFFWQWWMYVVIVALVVIVLMLLAYGYYWWKRRKLMRQLEALQSQNQPEVQRSL*
YANDHLDRNLLHSPTQPSSGYVADGFQTARPIVNGGAPATSGYVLASSPSVRFNSNTNGAPATAVPGRKT

>Ps_135399 (=PsAP8)
MQVAAILPMALSLSLATTAQAGDVLQQKLWGLSSGLAYYVQVNVGSQLYSSSSGSSASDSFNLLLDTGSANTAVVTADCCSLTN
EHLYSCSDSDTCVDQGTAVSVTYVSGSWSGEVVEDTFSGEGLGTVESMPFAEITAEDSFISSGYDGIVGLGYKAIASPSSDPPT
PYFDKVQSADGLSDVFSLQMCGALQALSLSNVSLADDSYLYAGEFVLGGTHGASGESFHKGDIVYTPLVQEKYYNVLVTDIGVN
GESLGLDCETINSPRSIIDSGTSNIAFPSSVYSAVIAELKTQVEKVTTDVSDSFFDDDSTCCSSECDPTNADSIIYSLPGLTIS
LAVDDDKSQQMTITIPAEYIWRPLVVSTGQGESACRVFGISEGSFTLLGDVFMDGLFTVHDRENERVGLAVADNCPNGVTSSKN
ITVETMTSEDSFC*SCVGSSDRKSSLLSSYVPFSGKPCFFWQWWMYVVLVAIVIIVLMLVAYGYYCWKRRKLLRQIEELRQSQNQ*
PRVQRSLYTNDRLDRNLLQTPTQPSSVYIVEAYTPPTAATVPIMNGGVPATNDYRLASSPSVRFNPNTTGAPATAAPGRKV

>Pr_78678 (=PrAP8)
MRAVASVVAMLLPLSLQTAQASDILQQKLWGLSSGLAYYVQVNVGSSLYSSSSGSSSSDSFNLLLDTGSANTAVVTAECCSLTN
EHLYSCSDSSTCVDQGTSVSVTYVSGSWSGQVVADTFSGQGLGTVESMPFAEITAEDSFISTGYDGIIGLGYKAIASPSSDPPT
PYFDTVKSADGLDDVFSLQMCGALQALSLSNVSMEDDSYLYAGEFLLGGTEGPSGERYHKGDIVYTPLVQDKYYNVIVTDIGVD
GESLGLDCETINSPRAIIDSGTSNLAFPSSVYSAVIAELKTQVEKVASDVSDSFFDDDSTCCDSECDPTNANSIIYSLPGLTIS
LAVDDEKSQQMTITIPAEYIWRPLVVSTGQGETACRVFGISEGDFTLLGDVFMDGLFTVHDRANDRVGLAVADNCPNGVTSSKN
ITVETMSAEDSFC*DCVGSTDRKSSLLSSYVPFSGKPCFFWQWWMYVVIVAIVIIILMAVAYGYFTWKRRKLMRQLEALQNQNQP*
QVQRNLYSNDRLDRNLLHTPTQPSSVYIVEAYTPPTSAVPIMNGGAPVTSGYRLATSPSVRFNPNTTGAPAAAVPGRKAYPS

>PITG_06900 (=PiAP9)
MTPRRRNSLLRCLALLLLGRETQAASAHYSLDLTGIASGTAYTLTVDIGTVDGSGSSSGNNAFRLIADTGSSNDAVLGTGCCGS
EAEVTYSCDASSTCTNAGGDVVTLAFAGANIQGQLMTDTWSSAEIGDISKTFLVIEKQDTFYRSTYDGITGLAYEALAASTGDT
VSSLYNVLVNTAKSTDAFGMLLCGTMQPMLQTGGTDFTHHSGQLLIGGTEGIDGETYYSGDMLYTPITREAWYVVTVTDIGYNG
ASLGFSCENYNEPQAIVDSGTSNLAFPSDVYNALMDQIKTATLEAIPDFDASYFDDSASCCDEDYCDPTSSSAALLQLPSIYFT
LGMETSDGSTSKHFTVEIPPEYYWRPEMNGDNSSMACRAIGISEGTSTVLGDVFMDGLYSYHDRVEGKIGLAVANNCPNNVTSS
KKVYTSEDSTDWC*SCFSSTLKKKSSWTTFLPWGSGCFFWLWWMYIVIASFVVVIACVGVLLWWHMTNKRMKKLQEEACRSNTSG*
RRTTRLATMQSSGFRGPALAPASPHNDYYVAPTSTPQRRSNRSGRSGSRHGSMKSPRSQDRRSKRKEKEMPIMAEPKYSTMSSP
RSPLSDTSSIALLSEPNSSVGSMESWKHKAKPYTPGNHNSNYKASYNDRWGASLKESEF

>Ps_135764 (=PsAP9)
MAALVTQRRLLLLLGCKLLLFGHEADAASAHYSIDLNGIASGTAYTLTVDIGSVEGSGSSGGSNAFRLIADTGSSNDAVLGTGC
CGSDAEVTYSCDASSTCSSSGSGSVTLSFAGANIQGQFMTDKWYSDEIGDITKSFLVIEEQDTFYRPTYDGITGLAYEALAASD
GSIQSLYNVLVDTGKTADSFGMLLCGTMQPMLQTGGTDFTLHSGQLLIGGTEGTEGESYYTGDMFYTPITREAWYVVTVTDIGY
DGSSLGLTCDKYNDPQAIIDSGTSNLAFPSDVYNALMDQIKSATLSAIPDFDETYFDDSTSCCDEDYCDPTSSNAALLELPSIY
FTLGMQTSDGGTSNHFTVEIPPEYYWRPEMNGKNASTPCRAIGISEGTSTVLGDVFMDGLYSYHDRADGKIGLAVAHNCPNEVN
STKKVYSSDDSDDWC*SCFSSTMKKKSSWATYVPWGSGCFFWQWWMYVVVASIFVVIACVCVLVWWHKTNKQMKKLQEDAYTAGS*
SGTRTTRLATLQSSGSGRGPALIPASPPNGYYAASASTPRRGSSRSRDRAGSRQRSPRSPRRKPKSREREVPIMVEPAYSNMSS
PRSPLSSTSSIALLSEPNSSLGSMESWKHRSKPYPAGHHQSYNHNHHHHGNNYNDRWGASLKENEF

>Pr_79884 (=PrAP9)
MAALELRRLLLLGCLLLLFRGKTKAASAHYSLNLTGIASGTAYTLTIDIGTLGGSSSGSNAFRLIADTGSSNDAVLGADCCGSD
ADVTYSCAASSTCTNSGGDKVTLSFAGANIQGQFMADTWSSSEIGDISKTFLVIEEQDTFYRSTYDGITGLAYEALAASAGETV
SSLYNVLVDTGGTTDAFGMLLCGTMQPMLQTGGTDFTHHSGQLMIGGTEGTNGETYYTGDMLYTPITRKAWYVITVTDIGYDGT
SLGLSCENYNDPQAIVDSGTSNLAFPSDVYNALMNQIKTATLAAIPDFDTSYFDDSASCCDEDYCDPTSSNAALLELPSIYFTL
GMETSDGSTSKHFTVEIPPEYYWRPEMNGDNSSTPCRAIGISEGSSTVLGDVFMDGLYSYHDRAGGKIGLAVADNCPNSVNSSK
KVYTSDDSDDWC*SCFSSTLKKKSTWTTYVPWGSGCFFWLWWMYVVLASVVIVIACICVVFWWHRTNEQMKKMREEAFGSGTSGG*
RTTRLATLQSSGSGRGPALVPASPPNSYYAAQTGTPKRGSNRSGSRRGSMRSPRSQEKRVKEKEKELPIMPEPKYFNMSSPRSP
LSSTSSIAILSETNSSLGSIEGWQQKSKPYPKGNRYNSGNNNHNDRWGASLKEDEF

>PITG_02623 (=PiAP10)
MVRVIAGFPAFLAIVSMLVEVEQVTGMTMELHRLPKHDVHPERYARRLSSEEDAPELVPLHLGLGTHYTWVYAGTPPQRASVIA
DTGSALMAFPCSGCDGCGHHTDQPFQAANSSTLVHITCAQKSLFQCKECHVQSDTCGISQSYMEGSSWKASVVEDIVYLGGESS
FDDKEMRNRYGTHFQFGCQSSEKGLFVTQVADGIMGLSNTENHIIAKLHRENKIASNLFSLCFTENGGTMSVGQPHKAAHRGEI
SYVKVIADRSAGHFYNVHMKDIRIGGKSINAKEEAYTRGHYIVDSGTTDSYLPRALKTEFLQMFKEIAGRDYQVGNSCKGFTNK
DLASLPTIQLVMEAYGDENAEVILDVPPEQYLLESNGAYCGGIYLSENSGGVIGANLMMNRDVIFDLGDQRVGFVDADCA*YAGA*
ANATAPPSIHKGGVATTTSTTNDTAPVVTPGAANTVAPTAATPAATTSTVVSTPKPTSAAVTSQPPTSTVEATVKPVSAIATAK
PVATAAAVPAAVAIKATKAPVVVATGSSTEDLNLQPLSGETTTTNQPSSSANTGEDKPKEKSSGTHPMVLTIVGAVLVVGFLLM
MLISVSRRRQKDGKEQLWSRVKGDEEDDDDDDEEEFGVVRNEKKKAVSAKHQRLDQEDDDDDDQSSSDEEDEVFDRISVQEESK
VDNHTLERL

>Ps_144093 (=PsAP10)
MVRVIASFPALAAASASLHALAEAMTMELHPLPKHSIHPDRYARRLNIEEDAPEIVPLHLGLGTHYTWVYAGTPPQRASVIADT
GSGLMAFPCSGCDGCGSHTDQPFQADNSSTLIHVTCSQQQSHFQCKECTEKSDTCAISQSYMEGSSWKASVVEDVVYLGGESSF
HDEAMRDRYGTHFQFGCQSSETGLFVTQVADGIMGLSNSDTHIVAKLHRENKIPSNLFSLCFTENGGTMSVGEPNTKAHRGEIS
YAKVIKDRSAGHFYNVNMKDIRIGGKSINAKEEAYTRGHYIVDSGTTDSYLPRAMKNEFLQVFKEVAGRDYQVGTSCHGYTNED
LASLPKIQLVMEAYGDENGEVIIDIPPEQYLLHNDNSYCGSIYLSENAGGVIGANLMMNRDVIFDNGNQRVGFVDADCA*YQGGN*
STKTTPPSIGDHITSSSNSSETTAVPAATAASNASVPASTSAVAGTTFASRAPTAAPTAAQTAAPATATTTAPKPVATPSATVE
TTPEPTSAEATSAPTATAASTPKVTEESIPEATSATATSEPVATAAATPATAVDEASIALDSASSNSVQNIQPLSEEATTKTQA
SSSADAADLTAKEKSSGTHPMVLTIVGAVLVVGFLLMMLISVSRRREKTGKEQLWSRVKGSEEDEDDDDEEEFGLVRNDKKGGS
TNHQRLDQEEDEDHHGQDSSSDEEDEVFDRKSMQEEAKVDNRTLERL

>Pr_77495 (=PrAP10)
MVHVYAGFPVLLAAISTITTLAEAAQSAGMTMELHRLPQHEIHPERYARRLNIEEDAPELVPLHLGLGTHYTWVYAGTPPQRAS
VIADTGSGLMAFPCSGCDGCGSHTDQPFMADNSSTLVHVTCSQQSFFQCKECKQTSDTCGISQSYMEGSSWKASVVEDVVYLGG
ETSFQDEEMRNRYGTHFQFGCQSSETGLFVTQVADGIMGLSNADNHIVAKLHRENKIPSNLFSLCFTDQGGTMSVGQANTNAHR
GEISYAKVISDRSASHFYNVHMKDIRIGGKSINAKEEAYTRGHYIVDSGTTDSYLPRALKTEFLQMFKEVAGRDYQVGTSCHGF
TNEDMASLPTIQLVMEAYGDENAEVVLDVPPEQYLLNANGAFCGSIYLSENSGGVIGANLMMNRDVIFDLGNQRVGFVDADCA*Y*
QSNSSTTASPSISNDSTSSTDGETNNVVPIVTNAATVPSATEATVESSPKPTSAAAASEPTVTAASTPEATVANISTLDLASST
ADLNIQPLSEEGTTKTQSSSSGVAADPKAKEKSSGGAHPMVLTIVGAVLVVGFLLMMLISVSRRRQKTGKDQLWSRVKGSEEDE
DDDDEEEFGMVRNDKKGSAKHQRLDADDDDDDQSSSDEEDEVFDRKSLQEETKVDNRTLERL

>PITG_02624 (=PiAP11)
MTSRCAASGVPWLAFLAAIAVVVCSLVSATVASEQSFLKIRLHKQQQQSSELSHRLAHQQARAHRRAQEAVGNSSGNSTRGALA
LSEAPLGVGYGTHYAEIHLGIPAQRASVIVDTGSHLTALPCSTCNGCGTHTDPLFDVSKSTTAKYLGCHDFDSCRSCENDRCII
SQSYMEGSMWHAIMVDELVWVGGFSTPSDEMEGILKTFGFRFPVGCQTKETGLFITQKENGIMGLGRHRSTVMSYMLNAGRVTQ
NLFTLCFAGDGGELVFGGVDYSHHTSDVGYTPLLDDKSAYYPVHVKDIRMNGVSLGIDAGTINSGRGVIVDSGTTDTFFDSKGS
RAFMKAFQNAAGREYSEKRMDLTADELAALPTISIILSGMKGDGTEDIQLDIPASSYLTPSDKVGSYNGNFHFSERSGGVLGAS
TMIGFDVIFDTENKRVGFAESDC*GKSYANTSTQTPIASNSTGQPIPQTEAPAASNTTTLGNTTQQPVSTTASSSSSSNNSSVES*
NTTTAALVDVTDSVDVSTSSSTGATTRRASPKFGAFVAEVILISLVGVALGVMVWTKWRTRSWSRIPNATETSRAHMETVVDMN
DNRSLSPTSPPGSPLSPRSRAARKGQSPKFTIGSPVDEEDADKFREEEEG

>Ps_158877 (=PsAP11)
MTPPRASGGRWRAFVAVLVAAICTQVTSLTASEQGLLKIQLHKQQQPSAELSYILAHQQARVQRRAQEAGNADGDSPVGAFALS
EAPLGVGYGTHYAEIYLGIPAQRASVIVDTGSHLTALPCSTCQGCGQHTDPLFDVSKSTTAKYLACHDFDSCRSCEQDRCYISQ
SYMEGSMWEAVMVDELVWVGGFSSPADEMEGVLKTFGFRFPVGCQTKETGLFITQKENGIMGLGRHRSTVMSYMLNAGRVTQNL
FTLCFAGDGGELVFGGVDYSHHTSDVGYTPLLSDKSAYYPVHVKDILLNGVSLGIDTGTINSGRGVIVDSGTTDTFFDGKGKRA
FMSAFSKAAGRDYSESRMKLTSEELAALPVISIILSGMKGDGTDDVQLDVPASQYLTPADDGKSYYGNFHFSERSGGVLGASAM
VGFDVIFDVENKRVGFAESDC*GRSYSNATTAAPIASDSTNQPAPATPVSVDSNATEQPAPSNTSVTSSSNSSSMDSNTTATALA*
NTVGSPDATTSISSTEGTTKRSSPPFGAFIAEVILISLVGVALAVMVWTKWRTRAWSRIPAESSRAHMQTIVEISESRSPSPSS
PPGSPLSPRSRAARKGPSPKFTIGSSGEEDGDELRDKEEGAGSPKVLTRPQGPR

>Pr_77493 (=PrAP11)
MTSRSGAAGARWRASLAVFAAVAYTQMTSLAAGKQSFLKIQLHKQQQPSADLAYRLATQQERARRRAQEETGNADGDFTLGTLA
LSEAPLGVGLGTHYAEIYLGIPAQRASVIVDTGSHLTALPCSTCDGCGTHTDPLFDVSKSTTAKYLGCHDFDSCRSCEQNRCYI
SQSYMEGSMWQAVMVDELVWVGGFSSKDDEVEGVLKTFGFRFPVGCQTKETGLFITQKENGIMGLGRHRSTVMSYMLSAGRITQ
NLFTLCFAGDGGELVFGGVDYSHHTSNVGYTPLLDDKSAYYPVHVKDIRLNGVSLGIDAGTINSGRGIIVDSGTTDTFFDAKGQ
RSFLNAFKNAAGGRAYNEKSMKLTTDELASLPTISIILSGMKGDGTDDIQLDVPASQYLTPSDDDGSYYGNFHFSERSGGVLGA
STMVGFDVIFDAENKRVGFAESDC*GKGYSNNSSATPIASDNTEQQTSESAAPITADTTEPQLLVNSSAASSSNNSFAESNATAT*
ALANTVGSPEVAASSTTGVATKRSSPAFGAFVAEVILISLVGVALAVMVWTKWRTRAWSRIPDAAETSRAYMQTIVDIIDNDSP
SPASPPGSPLSPRSRAARKGPSPKFTIGSSEEGDRDELREEEEGAEICFPRS

>PITG_11522 (=PiAP12)
MPLGAVVCYAALASLTLLETCATSLPDTIQLEMFNSMARSQAANALQGRYRYHRSIDENLQALVTTSDEDPPDIDARRNEETVE
MGLHSGSGSHTIQVTIGGQQRELIIDTGSGKTAFVCTGCNKCGNKRKHQPFIFTDNTTYLSCDQSMTPLSNIGEPPCVDCENGK
CKYGQTYIEGDHWTAYKASDVMQLSSSFEARIEFGCIYEQSGVFLDQPSDGIMGFSRHPDSIFEQFYRQKVTHSRIFSQCLAEG
GGLLTIGGVDLARHTEPVRYTPLRNTGYQYWTVTLLSVSVGDANNTVQVDRKEFNADRGCVLDSGTTFLYMPESTKQPFRLAWS
RAVGSFSFVPESNTFYFMTSKQVAALPDICFWFKNDVHICLPSSRYFALVGNGIYTGTIFFTAGPKATILGASVLEGHDVIYDV
DNHRVGIAEAMC*DQPLQAEVELSLDPGGDKFRASFDYSQAPQWMLACVTLLAVAGLINAIWVAAAIEGDTTVNIKGSSTAKESN*
AWQDEEFSFFLMMEDEPK

>Ps_141367 (=PsAP12)
MAVGALGRYAVLASLAVWGARAAVDAPNTIQLEMSNGVARSQAVNALQGRYRYHRSMDEELQAKQTLSARDPVDESSPRNEATV
ETALRSGSGSHTIQVLVGGQQRELIIDTGSGKTAFVCVGCNNCGSKRRHEPFVLTGNTTYLSCDRSMTLQTSWGEPACMACENG
KCKYGQTYVEGDHWSAYKASDMMQLSPSFEARIEFGCIYEQSGVFLDQPSDGIMGFSRHPDSIFEQFYRQKVTHSRIFSQCLTE
GGGMLTIGGVDLTRHTEPVRYTPLRSTGYQYWTVTLQSVSVGNQSNTLQVDTYEYNADRGCVLDSGTTFLYMPERTKEPFRLAW
SRAVGSFSYIPQSDTFYSMTPDQVAALPDICFWLKNDVHICLPPSRYFAQVGDGVYTGTIFFSPGPRATILGASVLEGHDIIYD
VDNNRVGIAEAMC*DQPMQAAVELSLDPGGEKFHAEFDYSQAPQWMFASITLLAVAGLINAIWVAAATQGEEIRPAGPTKPAQQT*
TTAVANDWQGEEFSFFLMQEDGDRQPC

>Pr_74839 (=PrAP12)
MALGVVGRYAVLVSLAMWGAYAAALSPDTIQLEMSNRMARTQVVNALQWRYRDRRTVDEDLQALQMVRDEELLGEDSTRNEATV
ETGLHSGSGSHTIQVSVGGQQRELIIDTGSGKTAFVCVGCNNCGSKRTHQPFVVTANTTYLSCDRSLTLSTGRGEPPCAACEDG
KCKYGQTYVEGDHWSAYKASDIMQLSASFEARIEFGCIYEQSGVFLDQPSDGIMGFSRHPDSIYEQFYRQKVTPSRIFSQCLTE
GGGLLTIGGVDLARHTEPVRYTPLRNTGYQYWTVTLLSVSVGNSNNTLQVDSSEYNADRGCVLDSGTTFLYMPESTKEPFRLAW
SRAVGSFSFVPMSDTFYTMTREQVAALPDICFWFKNDVHICLPSSRYLAQIGDGVYTGTIFFTPGPRATILGASVLEAHDIIYD
VDNHRVGIAEAMC*DQPLQTAVKLSLDPGGDKFHAEFDYSQAPQWMLAGVTLLAVAGLINAIWVAAATEGETPSVTGKATKAPPA*
SVTNDWQGDEFSFFLMQEDEQ
