# Supplementary material for: The aspartic proteinase family of three Phytophthora species
Source: BMC Genomics. 2011 May 20;12:254. doi: 10.1186/1471-2164-12-254 (PMC3116508; doi:10.1186/1471-2164-12-254)
Supplement: Additional file 5 — Signal peptides and propart segments at the N-terminal end of the mature enzyme regions of PxAP1-12. Gaps were introduced to optimize alignment. Each signal peptide and propart segment is spatially separated and marked by the respective boxes at the top. The lack of a signal peptide or propart segment in individual polypeptides is indicated by ------ none -------. [file 1471-2164-12-254-S5.PDF]

Signal peptides and propart segments at the N-terminal end of the mature enzyme regions of PxAP1-12.

```
               signal peptide                                          propart

PiAP1               MMLRCSVLCSLLLALSSAA  VMRVPMIKRSDDEFVSSLLHDVHAMQRPVVWSPDASPINNQNEGQSTVEG
PsAP1          MLRCSLLCSALLALALCASDAA  VLRVPMRKRSDDEFVASLLHDVHALNRPVVWAPEADAFPSPATDADAVQG
PrAP1                 MLRCSLLVSALLLASDAA  VLRVPMIKRSDDEFVSSLLRDVHALQRPAVWSPQEPNSDTFNPDVENVED
---------------------------------------------------------------------------------------

PiAP2        ----------(none)----------  MT-SMAVAKVPMRRGQTHWTRSRRLVSSASN
PsAP2        ----------(none)----------  MTTSKAVAKVPMRRGQSPWTRSRRLASSASN
PrAP2        ----------(none)----------  MAS-----KVPMRRAQSPWTRSRRLVSSDSN
---------------------------------------------------------------------------------------

PsAP3          MIHRWVV-LLACAVAVVCIAPCSA  MIRIPLSRRQRGGIELSEPAAAMQGLSTVTHT---HSK
PrAP3          MQWCECAALLVYLLIAVCAARCSA  VVRIPLSMRRRGISPQ---AAAMQELSTPTHSVSIHAK
---------------------------------------------------------------------------------------

PiAP4              MRLRLLP--SLAILSTHCVA  TGAEPLRVSLARRRPP---------TAATP-DWIQSEAVAYHHGDPATPDWIMTESNSTQDAAQPPLPHGKR
PsAP4              MRLRLLSLLTLLAPSQCAAS  DAGPLRVSLARRRSPPDTAPAVASATTATATDWIQSEARVYHTHAP-VPEWTVAEANATQDAAQPPLPTAKR
PrAP4              MWLHLL---TALALLTSQFA  HAAPFKLSLARR-LP-------------ATDWIQSEANAYRAH-PAAPEWTVAEANATQDAAQPPLPSAKR
---------------------------------------------------------------------------------------

PiAP5              MRLGLLVAALAALSFQPASS  LLRVPLTAKAQRRSADHLLQFHTQPSDAV-TNAVQADHVLQYAAINT--------QDILHNAQDLAA
PsAP5  MAATPGRRRLALALALLSLLSLTLQRVHSVAA  PLRVPLRAASKRRNAQKLLQFHTQPEPKAAAALNVD-GVVSSLRARQPEAAG------LHDAQQLAA
PrAP5        MLNRLRLAALLAAFVTLALPSQGANS  LLRIPLTATTQRCSAQKLLQFHTQPSDAVTTNAVEAEGVLSSLNVLQNGDNSLRAQDTLHNAQQLEA
---------------------------------------------------------------------------------------

PiAP6            MGPRRVRLAAMALSLFCPCANS  ---------------(none)---------------
PsAP6         MVPHRRLRHAAAGLALTLMGARAAA  ---------------(none)---------------
PrAP6         MARRRLRCAAVALSLAACASSPSAA  ---------------(none)---------------
---------------------------------------------------------------------------------------

PiAP7      MDRCKRILRYLIVLVLLLTQDFSAASQC  ---------------(none)---------------
PrAP7               MARQLYCWLVLVHAIAATS  ---------------(none)---------------
---------------------------------------------------------------------------------------

PiAP8                 MRIAVATALLTQTAQA  ---------------(none)---------------
PsAP8             MQVAAILPMALSLSLATTAQA  ---------------(none)---------------
PrAP8             MRAVASVVAMLLPLSLQTAQA  ---------------(none)---------------
---------------------------------------------------------------------------------------

PiAP9             MTPRRRNSLLRCLALLLLGRE  ---------------(none)---------------
PsAP9          MAALVTQRRLLLLLGCKLLLFGHE  ---------------(none)---------------
PrAP9           MAALELRRLLLLGCLLLLFRGKT  ---------------(none)---------------
---------------------------------------------------------------------------------------

PiAP10       MVRVIAGFPAFLAIVSMLVEVEQVTG  MTMELHRLPKHDVHPERYARRLSSEE
PsAP10         MVRVIASFPALAAASASLHALAEA  MTMELHPLPKHSIHPDRYARRLNIEE
PrAP10    MVHVYAGFPVLLAAISTITTLAEAAQSAG  MTMELHRLPQHEIHPERYARRLNIEE
---------------------------------------------------------------------------------------

PiAP11 MTSRCAASGVPWLAFLAAIAVVVCSLVSATVA  -SEQSFLKIRLHKQQQQSSELSHRLAHQQARAHRRAQEAVGNSSGNSTRGA
PsAP11  MTPPRASGGRWRAFVAVLVAAICTQVTSLTA  -SEQGLLKIQLHKQQQPSAELSYILAHQQARVQRRAQEA-GNADGDSPVGA
PrAP11  MTSRSGAAGARWRASLAVFAAVAYTQMTSLA  AGKQSFLKIQLHKQQQPSADLAYRLATQQERARRRAQEETGNADGDFTLGT
---------------------------------------------------------------------------------------

PiAP12         MPLGAVVCYAALASL-TLLETCAT  --SLPDTIQLEMFNSMARSQAANALQGRYRYHRSIDENLQALVTTSDEDPPDIDARR
PsAP12         MAVGALGRYAVLASLAVWGA--RA  AVDAPNTIQLEMSNGVARSQAVNALQGRYRYHRSMDEELQAKQTLSARDPVDESSPR
PrAP12         MALGVVGRYAVLVSLAMWGAYAAA  --LSPDTIQLEMSNRMARTQVVNALQWRYRDRRTVDEDLQALQMVRDEELLGEDSTR
```
