# Supplementary material for: The aspartic proteinase family of three Phytophthora species
Source: BMC Genomics. 2011 May 20;12:254. doi: 10.1186/1471-2164-12-254 (PMC3116508; doi:10.1186/1471-2164-12-254)
Supplement: Additional file 6 — Alignment in orthologous sets of the extensions at the C-terminal ends of the mature enzyme regions of PxAP2 - 12 detailed in Figures 2, 3, 4, 5. Gaps were introduced to optimize alignment. Hydrophobic residues contributing potential membrane-spanning stretches are boxed. [file 1471-2164-12-254-S6.PDF]

Alignment in orthologous sets of the extensions at the C-terminal ends of PxAP1 – 12.

```
PiAP2    TPGSQAVSLPASSDSSGDNTGGNGFGGGSG-GISGITFNDDDKSHLTTILAIVLGVLGFFLIIAVVTSLFRCMCR
PsAP2    TPGSQAVSLPASSDSSGDNTGGGGFGSGSD-GTSGTL-SSDDKSHLVTILAIVLGVLAFFFIIAVVTYLFRCICR
PrAp2    TPGSQAVSLPASSDSSGDNTGGNGFGGNSDGGTSGTSSSDDDKSHLVTILAIVLGVLAFFFIIAVVQYLFRCVCR

PiAP2    RRRASRHEQSLQPVLVVPGGYYQPQPAIQPSQGYYAQH
PsAP2    RKRASRHEQSLQPVLVVPGGYYQPQPAVQPSQGYYAQH
PrAP2    RKRASRHEQSLQPVMVVPGGYYQPQPAIQPSQGYYAQH
```

- -

```
PsAP3    GWSSHCRARLQLEEKSHVLLFVSNVNS
PrAP3    GSSPRCSIGEYKMPST
```

- -

```
PiAP4    GDVCQGGRGNIYGDNGEGGAFGAWENAFLLGSCFAAACMFLFVFLLNQQDEEPNLSQDEGFLGAKIDRR-NSPQG
PsAP4    GDICQGGRGNIYGDDGEGGAFGAWENAFLLGSCFAAACMFLFVFLLNQQDEEPYLNQDESFLGGIIDRRTNSPQG
PrAP4    GDICQGGRGNIYGDNGEGGAFGAWENAFLLGSCFAAACMFLFVFLLNQQDEEPNLNQDEGFLGALIDRRANSPSG

PiAP4    FQTHDPKRPLLYDDEESQHVYATSSSSTSQASSYAEARRNFAALPSP-RSSDGYQSDGSCVEV
PsAP4    YQVHDPKRPLLYDDE-DQHAYATSSTSTSQASSYAEARRSFAALPSPPGSTDGHQSDGSCVEV
PrAP4    YQTHDPKRPLLFDDEESQHVYATSSTTTSQASSYTEARRSFAALPSPPGSNDGHMSDGSCVEV
```

- -

```
PiAP5    NSTLCGVEDMQDKTVNSNGNDLLPSQ-KSPFFENSFNMYDMDTHAVLVLFLSGLSLVGSGFIVSSFVQYPILRSF
PsAP5    NSTLCGVEDIDE-TVSSDDTDGLPSKVSSPFFENSFNMYDMDTHAVLVLFLSGLSLVGSGFIVSSFVQYPILRSF
PrAP5    NSTLCGVEDFEKAAGDSNDNDLLPSKQASPFFENSFNMYDMDTHAVLVLFLSGLSLVGSTFIVSSFVQYPILRGF

PiAP5    RSFSIFFWLSVCTLGYNLTLWIAGVWRAHQTHVLFCALLKSSQQFFGTAILLFSAAVGLELIRAVRWTHSSTVEY
PsAP5    RSFSLFFWLSVCTLGYNLTLWIAGVWRAHQTHVFFCALLKSTQQFFGTAILLFSAAVGLELIRAVRWTHSSTVEY
PrAP5    RSFSLFFWLSVCTLGYNLTLWIAGVWRAHQTHVFFCALLKSTQQFFGTAILLFSAAVALELIRAVRWTHSSTVEY

PiAP5    KLVYHVVIWCSAAFTGAFSLLTGVVGFLPDGAGPCRACFVGHSPGWARVLLFYLPATLTLTFAGSAVYLASKGSA
PsAP5    KLVYHVVIWCSAAFTGAFSLLTGVIGFLPDGAGPCRACFVGHSPGWARVFLFYLPATLTLTFAATAVYLAYTSNA
PrAP5    KFVYHVVIWCSAACTGAFSLLTGVIGFLPDGAGPCRACFVGHSPGWARVFLFYLPATLTLTFAGTAVYLAYTGSS

PiAP5    GLSLPPQAERARRSSGQLLSSCVATVAALFLPTLFGWLQSFGADWVTSGFFLYLSELCFYSQGLLNALSWAFNPS
PsAP5    GQSLPPQAERARRSSGQLLSSCVATAAALFLPTFFGWLQAFGAEWVTTGFLLYLSELCFYSQGLLNALSWAFNPS
PrAP5    GQSLPPQADRARRSSGQLLSSCVATVAALLLPTLFGWLQAFGTDWVTSGFLLYLSELCFYSQGLLNALSWAFNPS

PiAP5    YRVARYRGSNAVGGEATRLMGPN
PsAP5    YRVARYRGNNATGGEGMRLMGPN
PrAP5    YRVARYRGANATGGEAMRLMGPN
```

- -

```
PiAP6    DSAT-----SSAVCSGGWAPKLQFHSAHFLDAEASAWRAGWVFWSRVYLGIGVLLLLVAFALLWLILVVPSSEVKKV
PsAP6    ESSASSSSGGANVCSGGWAPKLQFHSTHFSDAEASTWRAGWVFWSRVYLGVGVLLLVVAFALLWLILVVPSSEVEKV
PrAP6    EPSLL--SGESGVCSGGWAPKLQFHSTHFSDAEASAWRAGWVFWSRVYLGVGVLLLLVAFALLWLILVVPSSEVEKV

PiAP6    FNWFYGPSDKSRRRQQQ---TECKLLPTRRSSYQGQ----SSD----SNRDDETAEPDSEPESPPAPSRRKSVSFDV
PsAP6    LDWFFGPSERSRRRQQQQVSTDSKVASSARGSYQTLENGVPIGGG---DGEEAPVEPDAEPESPRSASRRRSVCYDV
PrAP6    LDWLCGPSSKSRRRQQQ-PPLDSKASSGCHGTPQALESGISSDNRDCSNAHEASEEPDSEPAAATAPSRRKSVCYDV
```

- -

```
PiAP7    SDDN-VCLGGLQPALDYHGKPYEQYSSVQQPCQLWAGLCAVATALAVFAGLRTLLKTLHADPRIYINNG
PrAP7    VDDNGVCPGGVQPALDYRGRPYESFEDRRQSSPSFVGLCAAATALALLAGLQMVL
```

```
PiAP8    DCVGSADRKSSLISSYVPFSGKPCFFWQWWMYVVIVALVVIVLMLLAYGYYWWKRRKLMRQLEAL-QSQNQPEVQRSL
PsAP8    SCVGSSDRKSSLLSSYVPFSGKPCFFWQWWMYVVLVAIVIIVLMLVAYGYYCWKRRKLLRQIEELRQSQNQPRVQRSL
PrAP8    DCVGSTDRKSSLLSSYVPFSGKPCFFWQWWMYVVIVAIVIIILMAVAYGYFTWKRRKLMRQLEAL-QNQNQPQVQRNL

PiAP8    YANDHLDRNLLHSPTQPSSGYVADGFQ--T--ARPIVNGGAPATSGYVLASSPSVRFNSNTNGAPATAVPGRKT
PsAP8    YTNDRLDRNLLQTPTQPSSVYIVEAYTPPTAATVPIMNGGVPATNDYRLASSPSVRFNPNTTGAPATAAPGRKV
PrAP8    YSNDRLDRNLLHTPTQPSSVYIVEAYTPPTS-AVPIMNGGAPVTSGYRLATSPSVRFNPNTTGAPAAAVPGRKAYPS
```

```
PiAP9    SCFSSTLKKKSSWTTFLPWGSGCFFWLWWMYIVIASFVVVIACVGVLLWWHMTNKRMKKLQEEACRSNTSGRRTT
PsAP9    SCFSSTMKKKSSWATYVPWGSGCFFWQWWMYVVVASIFVVIACVCVLVWWHKTNKQMKKLQEDAYTAGSSGTRTT
PrAP9    SCFSSTLKKKSTWTTYVPWGSGCFFWLWWMYVVLASVVIVIACICVVFWWHRTNEQMKKMREEAFGSGTSGGRTT

PiAP9    RLATMQSSGF-RGPALAPASPHNDYYVAPTSTPQRRSNRSGRSGSRHGS-MKSPRSQDRRSKRKEKEMPIMAEPK
PsAP9    RLATLQSSGSGRGPALIPASPPNGYYAASASTPRRGSSRS---RDRAGSRQRSPRSPRRKPKSREREVPIMVEPA
PrAP9    RLATLQSSGSGRGPALVPASPPNSYYAAQTGTPKRGSNRS---GSRRGS-MRSPRSQEKRVKEKEKELPIMPEPK

PiAP9    YSTMSSPRSPLSDTSSIALLSEPNSSVGSMESWKHKAKPYTPGNHNS------NYKASYNDRWGASLKESEF
PsAP9    YSNMSSPRSPLSSTSSIALLSEPNSSLGSMESWKHRSKPYPAGHHQSYNHNHHHHGNNYNDRWGASLKENEF
PrAP9    YFNMSSPRSPLSSTSSIAILSETNSSLGSIEGWQQKSKPYPKGNRYN------SGNNNHNDRWGASLKEDEF
```

```
PiAP10   YAGA-ANATAPPSIHKGGVATTTSTTNDTAPVVTPG---------------------------AANTVAPT-----AA
PsAP10   YQGGNSTKTTPPSIGDHITSSSNSSETTAVPAATAASNASVPASTSAVAGTTFASRAPTAAPTAAQTAAPATATTTAP
PrAP10   YQSN-SSTTASPSISNDSTSSTDGETNNVVPIVTNA----------------------------------------AT

PiAP10   TPAAT-TSTVVSTPKPTSAAVTSQPPT---STVEATVKPV----SAIATAKPVATAAAVPAAVAIKATKAPVVVATGS
PsAP10   KPVATPSATVETTPEPTSAEATSAPTATAASTPKVTEESIPEATSATATSEPVATAAATPATAV---DEASIALDSAS
PrAP10   VPSAT-EATVESSPKPTSAAAASEPTVTAASTPEATVANI-------------------------------STLDLAS

PiAP10   STEDLNLQPLSGETTTTNQPSSSANTGEDKPKEKSSG-THPMVLTIVGAVLVVGFLLMMLISVSRRRQKDGKEQLWSR
PsAP10   SNSVQNIQPLSEEATTKTQASSSADAADLTAKEKSSG-THPMVLTIVGAVLVVGFLLMMLISVSRRREKTGKEQLWSR
PrAP10   STADLNIQPLSEEGTTKTQSSSSGVAADPKAKEKSSGGAHPMVLTIVGAVLVVGFLLMMLISVSRRRQKTGKDQLWSR

PiAP10   VKGDEEDDDDDDEEEFGVVRNEKKKAVSAKHQRLDQEDDDDD--DQSSSDEEDEVFDRISVQEESKVDNHTLERL
PsAP10   VKGSEEDEDDDDEEEFGLVRNDKKG-GSTNHQRLDQEEDEDHHGQDSSSDEEDEVFDRKSMQEEAKVDNRTLERL
PrAP10   VKGSEEDEDDDDEEEFGMVRNDKKG--SAKHQRLDADDDDDD---QSSSDEEDEVFDRKSLQEETKVDNRTLERL
```

```
PiAP11   GKSYANTSTQTPIASNSTGQPIPQTEAPAASNTTTLGNTTQQPVSTTASSSSSSNNSSVESNTTTAALVDVTDSV
PsAP11   GRSYSNATTAAPIASDSTNQPAPATPVSVDSN------ATEQPAPSNTSVTSSSNSSSMDSNTTATALANTVGSP
PrAP11   GKGYSNNSSATPIASDNTEQQTSESAAPITAD------TTEPQLLVNSSAASSSNNSFAESNATATALANTVGSP

PiAP11   DVSTS-SSTGATTRRASPKFGAFVAEVILISLVGVALGVMVWTKWRTRSWSRIPNATETSRAHMETVVDMNDNRS
Psap11   DATTSISSTEGTTKRSSPPFGAFIAEVILISLVGVALAVMVWTKWRTRAWSRIP--AESSRAHMQTIVEISESRS
PrAP11   EVAAS-STTGVATKRSSPAFGAFVAEVILISLVGVALAVMVWTKWRTRAWSRIPDAAETSRAYMQTIVDIIDNDS

PiAP11   LSPTSPPGSPLSPRSRAARKGQSPKFTIGSPVDEEDADKFREEEEG
PsAP11   PSPSSPPGSPLSPRSRAARKGPSPKFTIGSS-GEEDGDELRDKEEGAGSPKVLTRPQGPR
PrAP11   PSPASPPGSPLSPRSRAARKGPSPKFTIGSS-EEGDRDELREEEEGAEICFPRS
```

```
PiAP12   DQPLQAEVELSLDPGGDKFRASFDYSQAPQWMLACVTLLAVAGLINAIWVAAAIEGDTTV---NIKG--SSTAKE
PsAP12   DQPMQAAVELSLDPGGEKFHAEFDYSQAPQWMFASITLLAVAGLINAIWVAAATQGEEIRPAGPTKPAQQTTTAV
PrAP12   DQPLQTAVKLSLDPGGDKFHAEFDYSQAPQWMLAGVTLLAVAGLINAIWVAAATEGETPSVTGK--ATKAPPASV

PiAP12   SNAWQDEEFSFFLMMEDEPK
PsAP12   ANDWQGEEFSFFLMQEDGDRQPC
PrAP12   TNDWQGDEFSFFLMQEDEQ
```
